# Solution-Processable Electronic-Grade 2D WTe_2_ Enabled by Synergistic Dual Ammonium Intercalation

**DOI:** 10.1021/acsnano.5c01224

**Published:** 2025-04-02

**Authors:** Hyejung Yang, Kevin Synnatschke, Jiho Yoon, Hossein Mirhosseini, Ilka M. Hermes, Xiaodong Li, Christof Neumann, Ahiud Morag, Andrey Turchanin, Thomas D. Kühne, Stuart S. P. Parkin, Sheng Yang, Ali Shaygan Nia, Xinliang Feng

**Affiliations:** † Center for Advancing Electronics Dresden (cfaed) and Faculty of Chemistry and Food Chemistry, Technische Universität Dresden, 01062 Dresden, Germany; ‡ Max Planck Institute for Microstructure Physics, D-06120 Halle (Saale), Germany; § Center for Advanced Systems Understanding (CASUS), 02826 Görlitz, Germany; ∥ Helmholtz-Zentrum Dresden-Rossendorf (HZDR), 01328 Dresden, Germany; ⊥ Institute of Artificial Intelligence, Chair of Computational System Sciences, Technische Universität Dresden, 01187 Dresden, Germany; # Leibniz-Institut für Polymerforschung Dresden e.V., Hohe Straße 6, 01069 Dresden, Germany; ∇ Institute of Physical Chemistry and Center for Energy and Environmental Chemistry Jena (CEEC Jena), Friedrich Schiller University Jena, Lessingstrasse 10, 07743 Jena, Germany; ○ Frontiers Science Center for Transformative Molecules, School of Chemistry and Chemical Engineering, Shanghai Jiao Tong University, 200240 Shanghai, China

**Keywords:** 2D nanomaterials, solution-processable, electrochemical exfoliation, WTe_2_, thin films

## Abstract

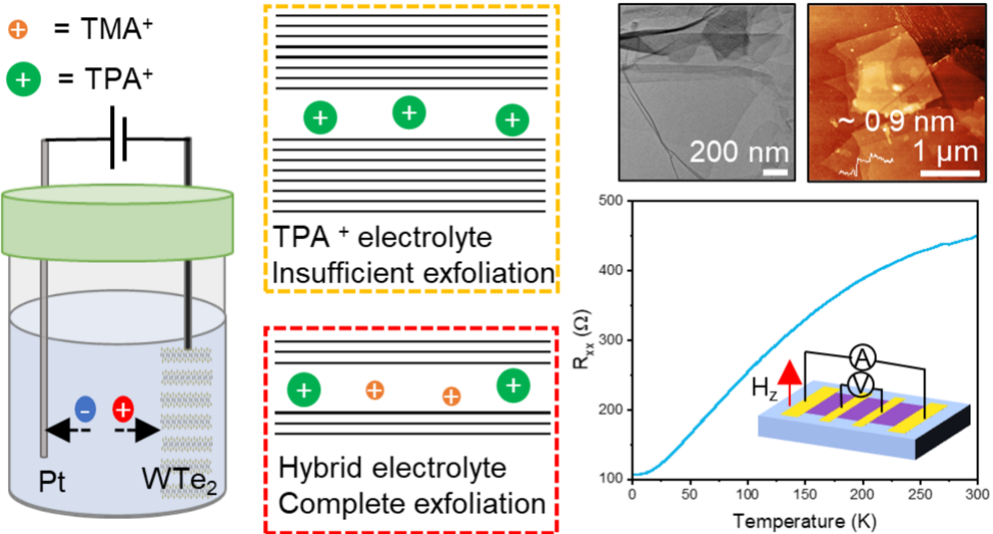

Tungsten ditelluride (WTe_2_) exhibits thickness-dependent properties, including magnetoresistance, ferroelectricity, and superconductivity, positioning it as an ideal candidate for nanoelectronics and spintronics. Therefore, the scalable synthesis of WTe_2_ with defined thicknesses down to the monolayer limit is crucial for unlocking these properties. Here, we introduce a universal electrolyte chemistry utilizing dual-ammonium compounds to exfoliate WTe_2_, enabling precise control over the intercalation stages and flake thicknesses. This approach achieves an 86% exfoliation yield, producing high-quality flakes averaging 2.83 nm in thickness, in which approximately 10% are monolayers. A solution-processed, single-flake device (10 nm thick) exhibits a magnetoresistance (MR) of 50% at 2 K and 9 T, and piezo-response force microscopy (PFM) indicates ferroelectricity in WTe_2_ flakes. Additionally, large-area WTe_2_ thin films (15 × 15 mm^2^), fabricated using Langmuir–Schaefer deposition, exhibit metallic behavior with a high conductivity of 2.9 × 10^4^ S/m. Overall, the hybrid electrolyte approach facilitates the scalable synthesis of high-quality, solution-processable, two-dimensional (2D) WTe_2_ flakes with excellent properties. This versatility of the developed method has been further exemplified through the exfoliation of other transition metal dichalcogenides (e.g., MoS_2_ and MoSe_2_), expanding the potential for the extensive application of exfoliated 2D materials in printable and flexible nanoelectronics.

Tellurium (Te)-based transition metal dichalcogenides (TMDs), including molybdenum ditelluride (MoTe_2_) and tungsten ditelluride (WTe_2_), have attracted significant recognition because of their diverse physical properties. MoTe_2_ exhibits semiconducting properties in the 2H phase (hexagonal structure)^1,2^ and shows ferroelectricity and superconductivity in the *T*_d_ phase (orthorhombic structure).^2,3^ WTe_2_, on the other hand, is a type-II weyl semimetal^4,5^ that exhibits an orthorhombic crystal structure in its energetically stable *T*_d_ phase.^6^ In addition, WTe_2_ shows giant, nonsaturating magnetoresistance in its bulk crystal form when exposed to a magnetic field.^7,8^ If the thickness is reduced to two or three layers, it displays ferroelectricity under an electric field, retaining bistable conductance from 4 K to room temperature.^9^ In monolayers, WTe_2_ shows topological insulator characteristics,^10^ enabling reversible and in situ electrostatic switching of superconductivity under an electric field.^11^ Moreover, quantum spin Hall effects are shown in WTe_2_ monolayers at temperatures up to 100 K,^12^ offering promising potential for quantum devices and topological field-effect transistors.^10,13^ These diverse physical properties make WTe_2_ an attractive candidate for advanced nanoelectronics, spintronics, and quantum computing applications.

To enable the potential applications of WTe_2_, it is essential to develop a scalable and efficient approach for producing high-quality, large-area WTe_2_ flakes ranging from monolayer to few-layer thickness. Among scalable synthesis methods, chemical vapor deposition (CVD) encounters difficulties in achieving stoichiometric growth of WTe_2_ because of the minimal difference in electronegativity and low chemical reactivity between tungsten (W) and tellurium (Te).^14,15^ Additionally, the CVD process primarily produces continuous thin films or substrate-bound crystals rather than dispersed, solution-processable flakes, which limits its compatibility with solution-based fabrication techniques commonly used for cost-effective electronic device manufacturing. In this regard, electrochemical exfoliation stands out as a promising method, offering both scalability and efficiency for producing high-quality few-layer flakes that are readily solution-processable.^16,17^ However, most electrochemical exfoliation studies on TMDs have focused on cathodic exfoliation and typically involve intercalating a single type of ammonium cations. This limited intercalation chemistry typically produces multilayer flakes (>5 layers), impeding the investigation of physical properties and potential applications with mono-, bi-, and trilayers.^16^ Alternatively, smaller alkali metal ions (e.g., Li^+^, Na^+^) intercalation can achieve low intercalation stage (i.e., stage 1 and 2), where a reduced number of host material layers are separated by the guest ions^18^ and can lead to thinner exfoliated flakes. However, these ions often induce phase transformations during the intercalation process due to the significant charge injection, which might alter the structural and electronic properties of the exfoliated flakes.^19−21^

In this study, we utilize dual-ammonium ion intercalation chemistry to precisely control the intercalation stages during electrochemical exfoliation, enabling the production of high-quality exfoliated two-dimensional (2D) WTe_2_ flakes (Figure 1a,b). Through molecular modeling, cyclic voltammetry (CV), and X-ray diffraction (XRD), we developed electrolytes from tetraalkylammonium tetrafluoroborate compounds ((C*_n_*H_2*n*+1_)_4_N^+^, BF_4_^–^, *n* = 1, 2···), achieving thin WTe_2_ flakes (1–4 layers) without phase transformations (Figure 1b). A single-flake device fabricated from these flakes demonstrated 50% magnetoresistance (MR) at 9 T and 2 K, highlighting the intrinsic magneto transport characteristics of WTe_2_. Moreover, ∼3.0 nm thick WTe_2_ flakes exhibited ferroelectric behaviors, as indicated by dual amplitude resonance tracking (DART) piezo-response force microscopy (PFM) switching spectroscopy. Additionally, the scalability potential of this approach was demonstrated by fabricating large-area WTe_2_ thin films (15 × 15 mm^2^) using Langmuir–Schaefer deposition. These films showed metallic behavior with a conductivity of 2.9 × 10^4^ S/m at room temperature, highlighting the potential for solution-processable, large-area device fabrication. Furthermore, the universality of this exfoliation method has been exemplified effectively with other transition metal dichalcogenides, including MoS_2_ and MoSe_2_, highlighting the broad applicability of producing high-quality 2D materials.

**Figure 1:**
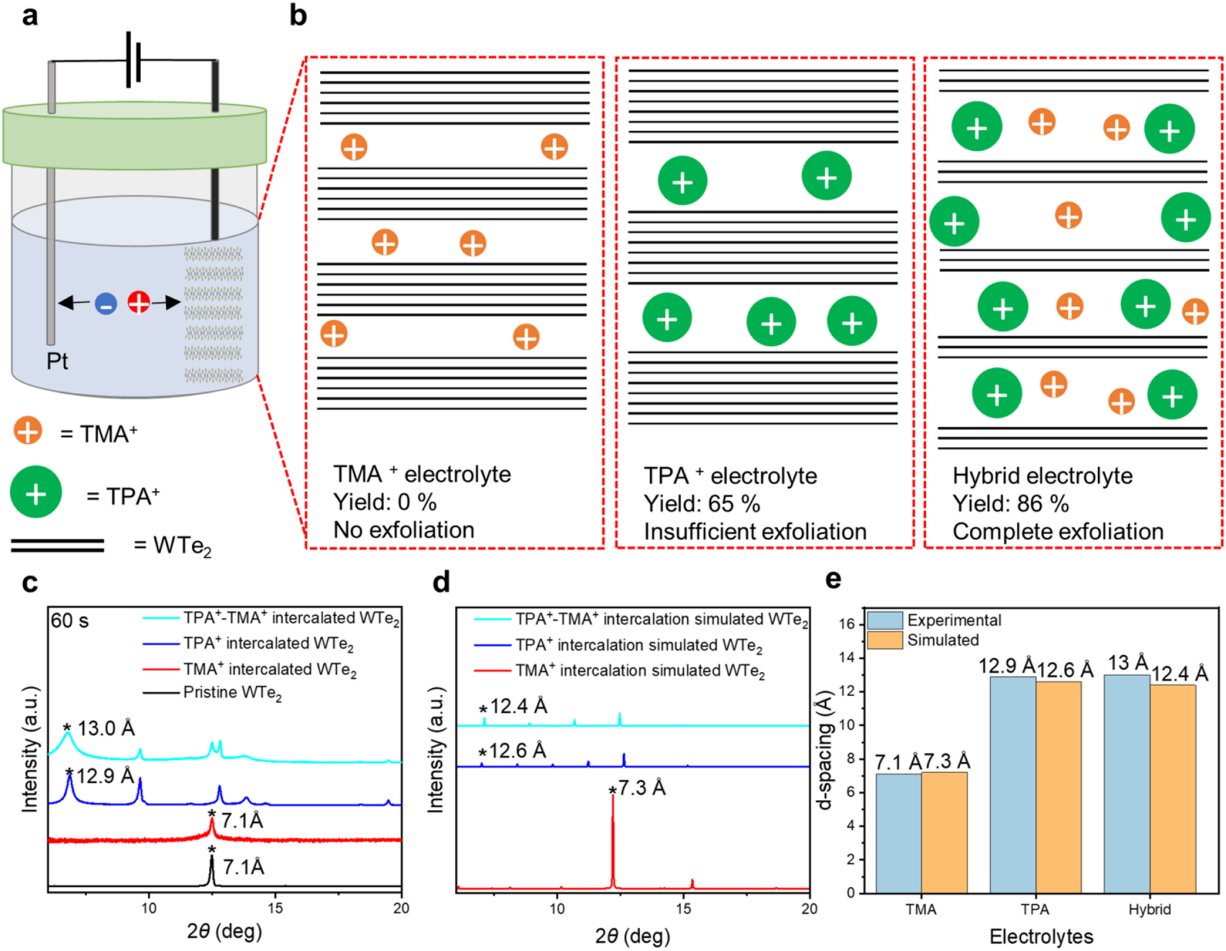
Interlayer distance engineering of WTe_2_ with different cations. (a) Electrochemical exfoliation setup. (b) Variations in the intercalation and exfoliation process for each electrolyte (TMA^+^, TPA^+^, and hybrid (TPA^+^-TMA^+^)) based on calculations and experiments. (c) XRD comparison at 60 s for pristine WTe_2_ and intercalated WTe_2_ with different cations. (d) Simulated diffraction patterns for each cation (TMA^+^, TPA^+^, and hybrid (TPA^+^-TMA^+^)) intercalation. (e) *d*-Spacing variations from experimental and simulated diffraction patterns for each cation (TMA^+^, TPA^+^, hybrid (TPA^+^-TMA^+^)) intercalation.

## Results and Discussion

### Dual Ammonium Electrolyte Design for Cathodic Electrochemical Exfoliation of WTe_2_

In this work, we employed cathodic electrochemical exfoliation, a widely used approach for intercalating quaternary ammonium cations into 2D van der Waals (vdW) materials under a negative potential. Building upon the conventional single-cation intercalation method, we first investigated tetrapropylammonium (TPA^+^) as a single cation, selected due to its diameter of approximately 7.4 Å,^22^ which closely matches the interlayer distance of WTe_2_ (7.07 Å),^23^ enabling effective intercalation.^24,25^ We then developed a hybrid electrolyte containing both TPA^+^ and tetramethylammonium (TMA^+^), combining two differently sized ammonium cations to improve exfoliation efficiency and achieve thinner flakes. This progression allowed us to compare the performance of the single-cation and dual-cation approaches.

**Figure 2:**
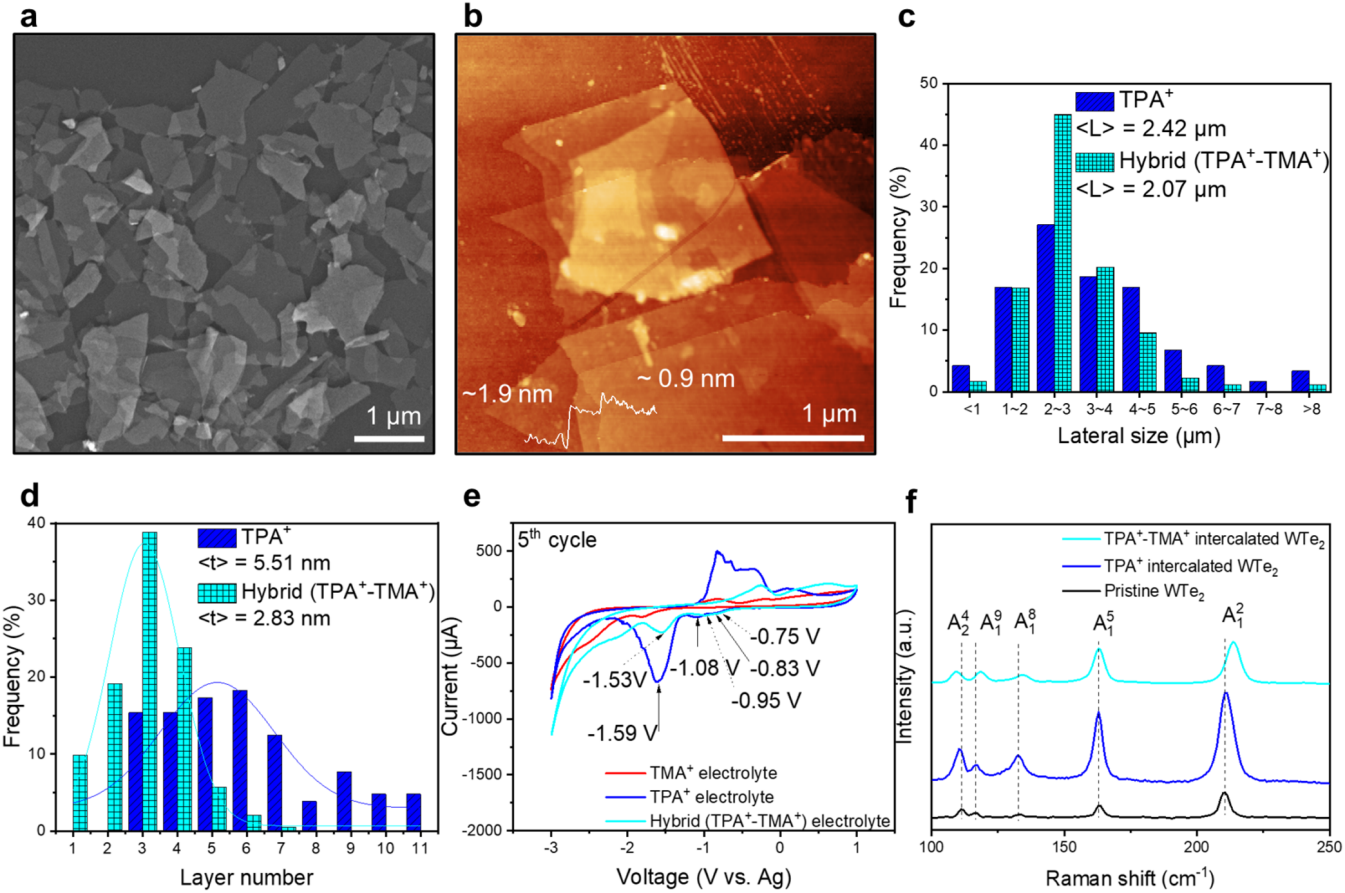
Characterizations of exfoliated WTe_2_ flakes. (a) Scanning electron microscopy (SEM) image and (b) atomic force microscopy (AFM) image of WTe_2_ flakes obtained using hybrid electrolyte (TPA^+^-TMA^+^). (c) Lateral size distribution and, (d) thickness distribution of exfoliated WTe_2_ flakes (normalized) obtained from TPA^+^ electrolyte and hybrid electrolyte. (e) CV analysis (fifth cycle) of each electrolyte (TMA^+^, TPA^+^, hybrid (TPA^+^-TMA^+^)). (f) Raman spectra of WTe_2_ flakes and crystals using a 532 nm laser.

The exfoliated WTe_2_ flakes obtained by TPA^+^ intercalation (TPA^+^ electrolyte) displayed a mean lateral size of 2.42 μm (Figures 2c, and S1a) and an average thickness of 5.51 nm (Figures 2d and S1b), which corresponds to more than five layers. This result suggests a high intercalation stage of TPA^+^ into the WTe_2_ vdW gaps.^26,27^ The intercalation stage refers to the degree of intercalation, indicating that the number of layers of host material are separated by inserted guest species.^18^ XRD measurement was performed after 60 s of TPA^+^ intercalation into the WTe_2_ crystal and showed a peak shift from 12.48° to 6.86°, indicating an expansion in interlayer distance from 7.1 Å to 12.9 Å (Figure 1c).

To gain further insights, diffraction patterns were calculated for different intercalation stage numbers of TPA^+^ and compared with the experimental XRD results. The best agreement with the experimental XRD pattern was obtained for the intercalation stage 8, where the interlayer distance increases from 7.07 Å to 12.6 Å (Figure 1d,e, Table S1 and Figure S2).

The confirmed intercalation stage 8 explains the thick (>5 layers) WTe_2_ flakes achieved during electrochemical exfoliation with TPA^+^ electrolyte. To explore the possibility of attaining a lower intercalation stage in WTe_2_ and thinner (<5 layers) WTe_2_ flakes, we performed XRD and CV analyses using shorter quaternary ammonium cations, including tetramethylammonium (TMA^+^, 5.6 Å) and tetraethylammonium (TEA^+^, 6.7 Å).^22^

From the CV analysis (at the fifth cycle), the TMA^+^ electrolyte showed intercalation peaks at around −1.78 V (Figures 2e and S5a), indicating successful intercalation of TMA^+^ in-between the vdWs gap of WTe_2_.^22,28,29^ In contrast, the TEA^+^ electrolyte did not show any noticeable intercalation peaks (Figure S5b). Under the same electrochemical conditions used for exfoliation (−2 V for 30 min, with each electrolyte concentration at 0.1 M), neither TMA^+^ nor TEA^+^ electrolyte resulted in exfoliation, likely due to the smaller diameter of these ions, which prevents efficient intercalation into the vdWs gap of the WTe_2_ crystal.^17^

Like TPA^+^, the diffraction patterns were calculated for TMA^+^ intercalated WTe_2_, and the simulated diffraction pattern of intercalation stage 6 is in reasonable agreement with the experimental XRD pattern (Figures 1d,e, Table S1 and Figure S3). This result highlights that while TMA^+^ intercalates into the vdW gaps of the WTe_2_ crystal, due to its smaller size, it will facilitate limited exfoliation, as evidenced by the consistent interlayer distance observed in the experimental XRD pattern (Figure 1c).

Based on these findings, we hypothesized that in a hybrid electrolyte composed of TMA^+^ and TPA^+^, TMA^+^ could increase the interlayer distance at the edge of the crystal, slightly widening the gap and reducing the intercalation barrier. This adjustment would facilitate easier intercalation of TPA^+^. To test our hypothesis, we calculated the diffraction patterns for WTe_2_ intercalated with both TMA^+^ and TPA^+^ for different intercalation stages and compared the results to the experimental XRD pattern. The best agreement with the experimental XRD pattern was obtained for the intercalation stage 3, where the interlayer distance increases from 7.07 to 12.4 Å (Figure 1b–d). This finding aligns with the measured XRD, where the interlayer distance increases from 7.1 to 13 Å (Figure 1c).

Furthermore, we performed CV analysis to gain deeper insight into the hybrid electrolyte intercalation mechanism.^22,28,29^ In the first cycle, the initial intercalation peaks for both electrolytes (hybrid and TPA^+^) were observed at around −1.9 V, indicating the activation of cation intercalation into WTe_2_ crystals (Figure S4). By the fifth cycle, the hybrid electrolyte showed an initial intercalation peak at −0.75 V, followed by −0.95 and −1.53 V (Figures 2e, and S5g). In comparison, the TPA^+^ electrolyte exhibited intercalation peaks starting at −0.83 V, followed by −1.08 and −1.59 V (Figures 2e, and S5c). The higher initial intercalation voltage in the hybrid electrolyte (−0.75 V) compared to the TPA^+^ electrolyte (−0.83 V) suggested that TMA^+^ increases the interlayer distances of WTe_2_, facilitating easier intercalation for TPA^+^.

In addition to the CV analysis, the distribution of relaxation time (DRT) obtained from electrochemical impedance spectroscopy (EIS) measurements indicated that diffusion is the dominant process in our system across all electrolytes.^30^ All electrolytes show a similar behavior for the diffusion barrier before intercalation. However, after intercalation, there was a notable decrease in the diffusion barrier in the hybrid electrolyte compared to the TPA^+^ electrolyte and TMA^+^ electrolyte (Figure S6). Notably, the hybrid electrolyte demonstrates more favorable kinetics for the diffusion process, suggesting a synergetic effect of combining two different cations, which reduces the intercalation stage and facilitates more efficient ion intercalation into the crystal lattice.

The reduction in the intercalation stage from TPA^+^ electrolyte (i.e., stage 8) to hybrid (TPA^+^-TMA^+^) electrolyte (i.e., stage 3) was further supported by statistical analysis of atomic force microscopy (AFM) measurements. With the TPA^+^ electrolyte, the mean thickness of the flakes is 5.51 nm (Figure 2d). However, upon use of the hybrid electrolyte, the mean thickness of the flakes is reduced to 2.83 nm (Figure 2b,d). Despite the reduction in thickness, the mean lateral size remained nearly constant from 2.42 μm (TPA^+^ electrolyte) (Figure 2c) to 2.07 μm (hybrid electrolyte) (Figure 2a,c) and could reach up to 20.8 μm (Figure S7). The aspect ratio (mean lateral size/thickness)^31^ increased from 439 using the TPA^+^ electrolyte to 731 with the hybrid electrolyte. Furthermore, the exfoliation yield improved from 65% with the TPA^+^ electrolyte to 86% with the hybrid electrolyte (Figure S8). This approach has also been employed in other 2D materials (e.g., MoS_2_ and MoSe_2_), showing consistent trends in terms of mean lateral size and thickness when transitioning from TPA^+^ to a hybrid electrolyte (Figure S9). This confirms the broad applicability of the hybrid electrolyte, which maintains a similar lateral size while achieving a narrower thickness distribution (Table S2). Furthermore, the electrochemical exfoliation process is highly efficient, completing within 30 min, highlighting its potential for scalable production (Table S3).

Raman spectroscopy was performed on both bulk WTe_2_ and exfoliated few-layer WTe_2_ flakes by using a 532 nm laser to assess the quality of exfoliated WTe_2_ flakes. Bulk WTe_2_ exhibited five Raman modes approximately at 110, 116, 132, 162, and 210 cm^–1^, which correspond to *A*_2_^4^, *A*_1_^9^, *A*_1_^8^, *A*_1_^5^, and *A*_1_^2^ modes at room temperature (Figure 2f).^23,32,33^ In the exfoliated flakes, the *A*_2_^4^ peak showed a shift to a lower wavenumber (red shift), while the other Raman-active modes shifted to higher wavenumbers (blue shift) as the layer thickness decreased. However, *A*_1_^5^ remained unaffected by the layer thickness, indicating its independence from the number of layers.^23,32^ The Raman spectra confirm that the exfoliated WTe_2_ flakes retain a comparatively high crystal quality throughout the exfoliation process, ensuring suitability for further investigations and applications.

**Figure 3:**
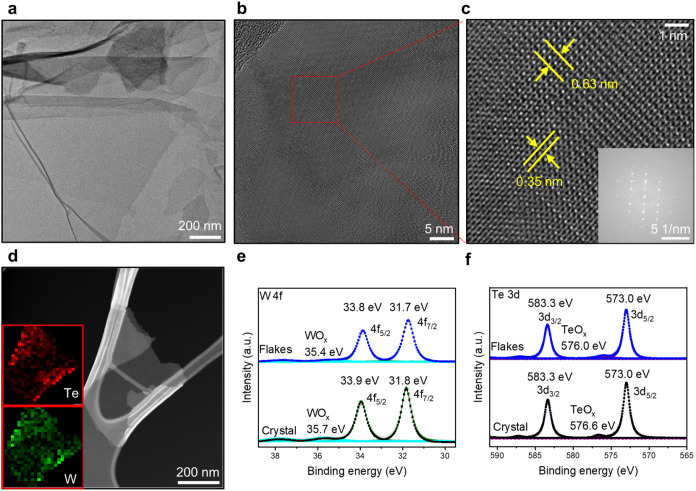
Characterization of exfoliated WTe_2_ flakes via a hybrid electrolyte. (a) TEM image of WTe_2_ flakes. (b) High-resolution TEM image. (c) Zoomed-in image of the HR-TEM image and FFT. (d) Element mapping of WTe_2_ flakes. (e, f) High-resolution XPS of WTe_2_ flakes and crystal.

After the successful high-yield (∼86%) exfoliation of the WTe_2_ crystal into flakes with the hybrid electrolyte, a comprehensive characterization of WTe_2_ flakes was accomplished by transmission electron microscopy (TEM) (Figure 3a) and X-ray photoelectron spectroscopy (XPS) (Figure 3e,f). TEM images showed that the thin flakes are flexible and sometimes are folded. High-resolution TEM (HR-TEM) images of different flakes were recorded (Figures 3b,c, and S10) to acquire detailed structural information. They showed the lattice constant of *a* = 0.35 nm and *b* = 0.63 nm, which agrees with the previous literature reports.^34,35^ Fast Fourier transform (FFT) was measured while obtaining HR-TEM images, confirming the rectangular shape of the unit cell (Figure 3b,c).^36^ Element mapping of WTe_2_ flakes (Figure 3d) confirmed the presence of W and Te atoms within the flakes. The intensity variation in mapping was attributed to the overlapping flakes on the TEM grid.

The surface composition and oxidation state of WTe_2_ flakes were also assessed by XPS, providing insights into the elements’ chemical bonding and oxidation states. For the high-resolution XPS of W 4f, 33.8 and 31.7 eV are assigned to W 4f_5/2_ and 4f_7/2_ peaks, respectively, representing W–Te bonds (Figure 3e). In the high-resolution XPS of Te 3d, 583.3 and 573 eV are referred to as the Te 3d_3/2_ and 3d_5/2_ peaks in W–Te bonds, respectively (Figure 3f).^36^ Additionally, XPS revealed the presence of oxidized species in the WTe_2_ flakes, manifesting as a TeO_*x*_ peak at 576.0 eV and a WO_*x*_ peak at 35.4 eV (TeO_*x*_, 10%, and WO_*x*_, 8%). These oxide peaks are comparable to those observed in pristine WTe_2_ crystals (Figure S11, TeO_*x*_, 5%, and WO_*x*_, 13%), suggesting that the exfoliation process does not significantly affect the purity or structural integrity of bulk crystals. Moreover, the exfoliated WTe_2_ flakes demonstrated good stability for 14 days under ambient conditions, with only a minor change in thickness from 6.1 to 6.3 nm (Figure S12).

### Intrinsic Property Measurement: Magnetoresistance and Ferroelectricity

**Figure 4:**
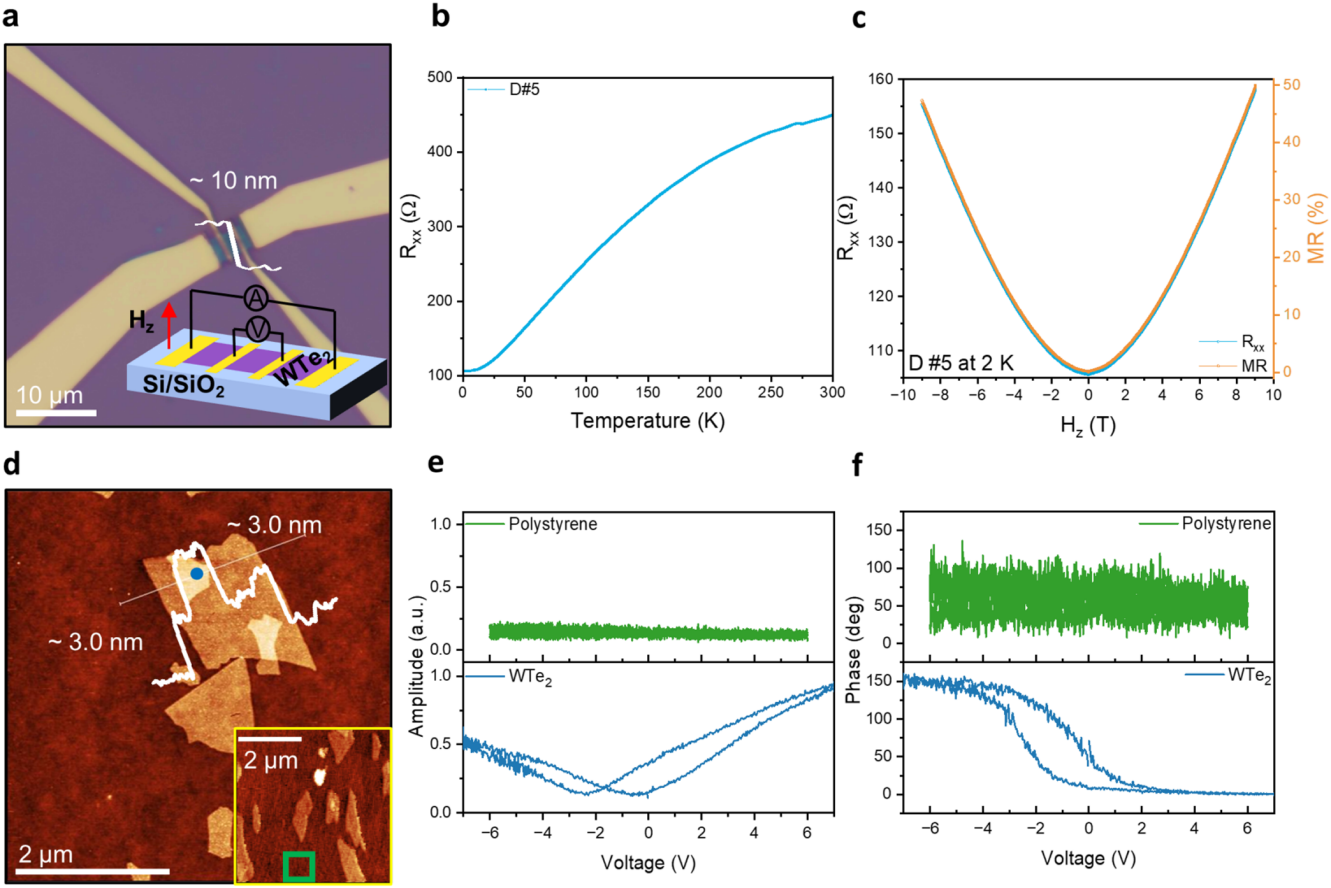
Magnetoresistance and ferroelectricity measurements of the WTe_2_ flakes. (a) Optical microscopy (OM) image of a single-flake device based on a 10 nm WTe_2_ flake. (b) Temperature-dependent longitudinal resistance (R_*xx*_). (c) Magnetoresistance as a function of the magnetic field at 2 K, and the magnetic field is applied in the c-direction. (d) AFM topography of WTe_2_ flakes on a gold-coated silicon wafer with an insulating polystyrene interlayer used for DART PFM switching spectroscopy. (e) PFM amplitude butterfly loops and (f) PFM phase hysteresis loops on the WTe_2_ flake and the polystyrene interlayer (green square of inset AFM image).

Magnetoresistance (MR) and ferroelectricity were measured to assess the intrinsic physical properties of the developed WTe_2_ flakes for nanoelectronics and spintronic applications. MR was measured on a device fabricated via solution processing, utilizing a 10 nm WTe_2_ flake (Figure 4a), and tested under varying temperatures and magnetic fields. A decrease in resistance (*R*_*xx*_) was observed as the temperature was lowered from 300 to 2 K, confirming the metallic nature of single-flake WTe_2_ (Figure 4b).^37^ At 2 K, MR reached the highest value, around 50% at 9 T (Figure 4c), which is comparable to or even better than the values previously reported for similarly thick WTe_2_ samples: around 3% for the 9.4 nm CVD-grown sample at 2 K and 9 T and approximately 80% for the 10 nm micromechanically exfoliated sample at 0.03 K and 10 T (Table S4). These findings indicate that electrochemical exfoliated WTe_2_ flakes exhibit intrinsic magnetoresistive properties, further confirming their high quality, structural integrity, and potential for applications in flexible and printable nanoelectronics and other further applications.

To further demonstrate the physical properties of WTe_2_ flakes maintained via electrochemical exfoliation with a dual ammonium intercalation approach, the ferroelectric characteristics of WTe_2_ flakes were investigated. Previous reports have suggested the presence of room-temperature ferroelectricity in the micromechanically exfoliated bilayer and trilayer WTe_2_.^9,38^ Here, dual amplitude resonance tracking (DART) PFM imaging and switching spectroscopy were performed on WTe_2_ flakes deposited onto both a gold substrate and a gold substrate with a polystyrene interlayer (polystyrene/gold substrate), respectively. The polystyrene interlayer was implemented to prevent leakage currents that could interfere with PFM switching spectroscopy (∼77.8 nm, Figure S13). DART PFM imaging on WTe_2_ flake on gold showed an increase in the PFM amplitude between the sample and the substrate (Figure S14c), suggesting the presence of a nonzero piezoelectric response, while the PFM phase of the flake was uniform, which indicates the absence of oppositely polarized out-of-plane domains (Figure S14b). To further probe the ferroelectric properties of WTe_2_ flakes, DART PFM switching spectroscopy was employed. The AFM topography of WTe_2_ flakes (∼3 nm thick, trilayer) on the polystyrene/gold used for PFM measurement is shown in Figure 4d. The PFM amplitude on the WTe_2_ flake exhibited the characteristic butterfly-shaped loops (Figures 4e, and S15b), while the PFM phase demonstrated a hysteresis with switching voltages of −0.38 and −2.4 V (Figures 4f and S15c), indicating ferroelectric behavior, correlate with previous observations.^9,38−40^ As a control, DART PFM switching spectroscopy on the polystyrene/gold substrate showed no hysteresis within the applied switching voltage ranges (Figure 4d,e).

**Figure 5:**
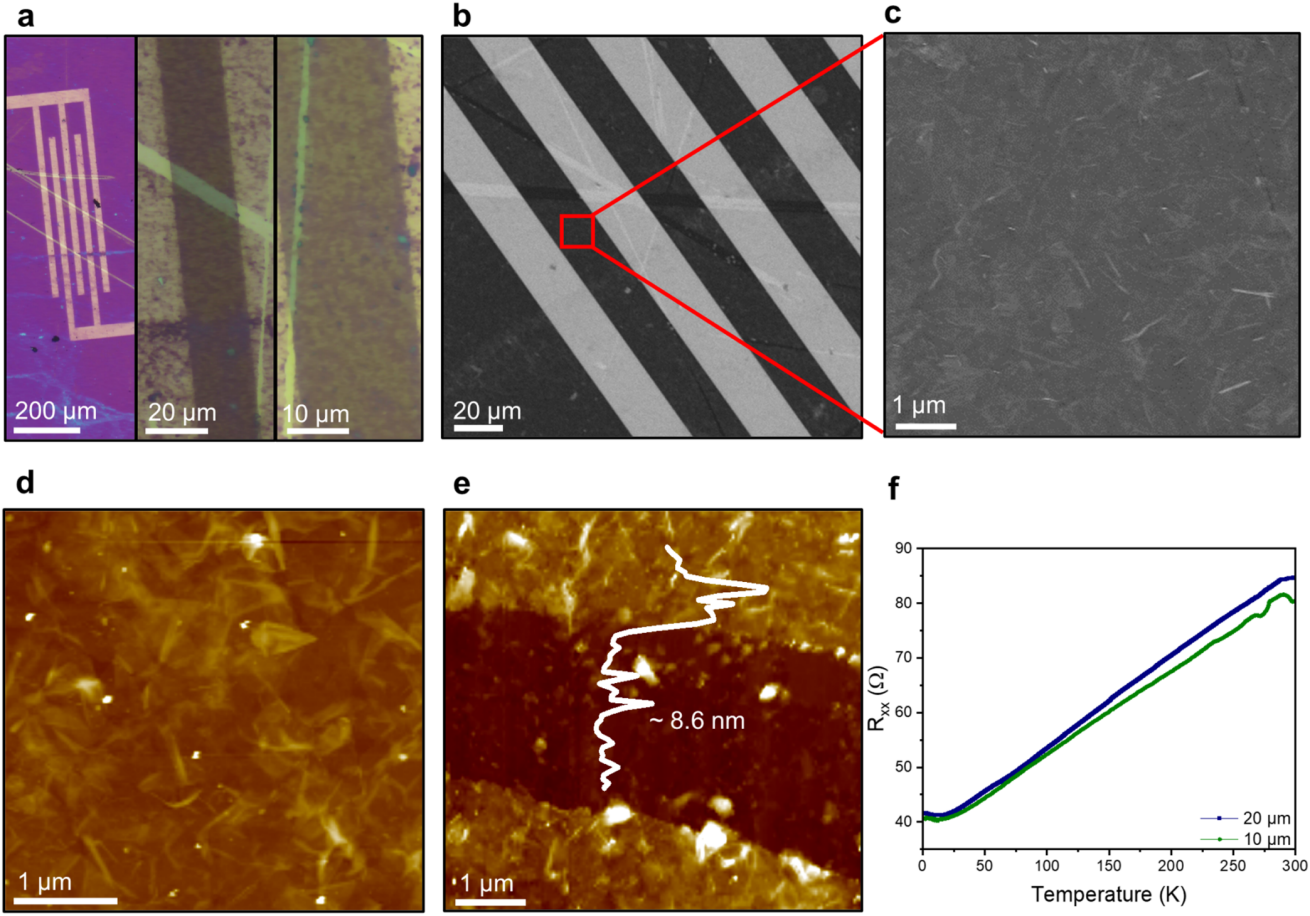
WTe_2_ thin film fabrication via the Langmuir–Schaefer deposition process. (a) OM image and (b) SEM image of WTe_2_ thin films. (c) Zoomed-in image of the SEM image. (d) AFM topography and (e) thickness of thin films. (f) Temperature-dependent longitudinal resistance (*R*_*xx*_).

Next, WTe_2_ thin films (15 × 15 mm^2^) were prepared by Langmuir–Schaefer approaches^41^ upon spreading the WTe_2_ inks/dispersions at the liquid–air interface and transferring the 2D flakes to different substrates, including quartz and Si/SiO_2_ substrates. Figure 5a–c illustrates optical microscopy (OM) images and scanning electron microscopy (SEM) images of WTe_2_ thin films. The AFM topography of the deposited thin film is depicted (Figure 5d), having a roughness *R*_q_ of 6.22 nm and a thin film thickness of approximately 8.6 nm (Figure 5e). These WTe_2_ thin films exhibited metallic properties, as indicated by decreased resistance with a decreasing temperature, as shown in Figure 5f. The conductivity of the WTe_2_ thin films reached 2.9 × 10^4^ S/m at room temperature, comparable to the conductivity of other solution-processed 2D materials, including graphene, PtSe_2,_ and MgB_2_ (Table S5). The results highlight the suitability of these high-quality 2D flakes for integration into flexible printed nanoelectronic systems, including the fabrication of large-area devices.

## Conclusions

In summary, we successfully produced high-quality WTe_2_ flakes via electrochemical exfoliation using a hybrid electrolyte consisting of tetramethylammonium (TMA^+^) and tetrapropylammonium (TPA^+^) cations. This approach achieved a high exfoliation yield (86%), producing flakes with uniform thicknesses ranging from monolayer to trilayer. Moreover, the exfoliated WTe_2_ flakes demonstrated intrinsic magneto transport properties with a magnetoresistance of 50% at 2 K and 9 T. Additionally, ferroelectric properties were characterized by DART PFM switching spectroscopy. Large-area thin films (15 × 15 mm^2^) were fabricated using the Langmuir–Schaefer method, exhibiting metallic behavior with an excellent conductivity of 2.9 × 10^4^ S/m, comparable to the thin film conductivity of other solution-processed 2D materials. In addition to WTe_2_, the exfoliation strategy was successfully applied to other TMDs (e.g., MoS_2_ and MoSe_2_), further demonstrating the versatility of the approach. This efficient and controlled synthesis enables the production of solution-processable 2D materials with few-layer thicknesses (1–4 layers), allowing access to thickness-dependent properties. These high-quality 2D materials show significant promise for use in cost-effective, scalable, and versatile applications in printed flexible electronic systems.

## Methods/Experimental Section

### Preparation of WTe_2_ Flakes

Few-layer WTe_2_ flakes were prepared via cathodic electrochemical exfoliation using a three-electrode setup, using a WTe_2_ crystal in Pt gauze (working electrode), Pt foil (counter electrode), and Ag wire (pseudoreference). Electrolytes were based on an anhydrous propylene carbonate (PC) with 0.1 M TPA·BF_4_, 0.1 M TMA·BF_4_, or a hybrid mixture (0.1 M TPA·BF_4_ + 0.01 M TMA·BF_4_). A constant voltage of −2 V was applied for intercalation for 30 min under argon. After intercalation/exfoliation, the flakes were collected, washed with PC and N,N-dimethylformamide (DMF), and redispersed in DMF via bath sonication.

### Device Fabrication and Magneto Transport Measurements

WTe_2_ dispersion (0.10 g/L in DMF) was spin-coated onto p-doped Si/SiO_2_ (285 nm) substrates and annealed at 200 °C for 10 h in argon. Electrical transport measurements were performed using Quantum Design PPMS DynaCool system (1.8–300 K, up to 9 T). Magnetoresistance (MR (%)) was calculated using MR (%) = (*R*_*xx*_(*B*) – *R*_*xx*_ (0)/*R*_*xx*_ (0)) × 100, where *R*_*xx*_ (*B*) is the longitudinal resistance with an applied magnetic field in the out-of-plane direction and *R*_*xx*_ (0) is the resistance at zero field.

### Thin Film Deposition

Langmuir–Schaefer deposition was used to fabricate uniform WTe_2_ thin films (15 × 15 mm^2^). Exfoliated inks (0.6 g/L in DMF) were deposited at the liquid–liquid interface under argon. Films were predried at 50 °C and annealed at 130 °C to enhance film uniformity and adhesion. For ferroelectric measurements, exfoliated flakes were spin-coated onto polystyrene-coated Au/Si substrates and characterized by dual-frequency DART PFM.

### Characterization Techniques

Flake morphology and thickness were analyzed by AFM (Parksystems NX10), SEM (ZEISS Sigma), and TEM (Carl Zeiss Libra 200). Structural properties were examined by using Raman spectroscopy (532 nm), XRD (Malvern PANalytical), and XPS (Thermo Fisher K-Alpha). PFM measurements were conducted with an Asylum MFP3D in DART mode to probe ferroelectric switching behavior.

Detailed information on all methods and characterizations is provided in the Supporting Information.

## Supporting Information Available

Experimental methods; additional material characterization; thin film preparation deposition procedure; magneto transport calculation details; and literature comparison (PDF)
