# Supplementary material for: Solution-Processable Electronic-Grade 2D WTe2 Enabled by Synergistic Dual Ammonium Intercalation
Source: ACS Nano. 2025 Apr 2;19(14):14309–17. doi: 10.1021/acsnano.5c01224 (PMC12004911; doi:10.1021/acsnano.5c01224)
Supplement: Supplementary file 1 — nn5c01224_si_001.pdf [file nn5c01224_si_001.pdf]

Supporting Information for

Solution-processable electronic-grade 2D WTe_2 enabled by synergistic dual ammonium intercalation

Hyejung Yang[1], Kevin Synnatschke[1], Jiho Yoon[2], Hossein Mirhosseini[3,4], Ilka M. Hermes[6], Xiaodong Li[1,2], Christof Neumann[7], Ahiud Morag[1,2], Andrey Turchanin[7], Thomas D Kühne[3,4,5], Stuart S.P. Parkin[2], Sheng Yang*[8], Ali Shaygan Nia*[1,2], Xinliang Feng*[1,2]

[1]Center for Advancing Electronics Dresden (cfaed) and Faculty of Chemistry and Food Chemistry, Technische Universität Dresden, 01062 Dresden, Germany

[2]Max Planck Institute for Microstructure Physics, Halle (Saale) D-06120, Germany

[3]Center for Advanced Systems Understanding (CASUS), 02826 Görlitz, Germany

[4]Helmholtz-Zentrum Dresden-Rossendorf (HZDR), 01328 Dresden, Germany

[5]Institute of Artificial Intelligence, Chair of Computational System Sciences, Technische Universität Dresden, 01187 Dresden, Germany

[6]Leibniz-Institut für Polymerforschung Dresden e.V., Hohe Straße 6, 01069 Dresden, Germany

[7]Institute of Physical Chemistry and Center for Energy and Environmental Chemistry Jena (CEEC Jena), Friedrich Schiller University Jena, Lessingstrasse 10, 07743 Jena, Germany

[8]Frontiers Science Center for Transformative Molecules, School of Chemistry and Chemical Engineering, Shanghai Jiao Tong University, 200240 Shanghai, China

*E-mail: xinliang.feng@tu-dresden.de

*E-mail: ali.shaygan_nia@tu-dresden.de

*E-mail: sheng.yang@sjtu.edu.cn

Table of Contents:

1. Experimental Section
2. Tables S1- S5.
3. Figures S1-S15.
4. References.

Experimental Section

Synthesis of WTe_2 flakes

WTe_2 flakes were produced by cathodic electrochemical exfoliation using a three-electrode system. In this setup, a WTe_2 crystal (HQ graphene) confined on platinum gauze (Alfa Aesar) was used as the working electrode, platinum foil (Alfa Aesar) was set up as the counter electrode, and an Ag wire was employed as a pseudo-reference electrode. The working and counter electrodes were aligned in a parallel arrangement, maintaining a 2 cm distance. The electrolyte was prepared by mixing tetrapropylammonium tetrafluoroborate (TPA·BF_4, Sigma-Aldrich, 98%) and tetramethylammonium tetrafluoroborate (TMA·BF_4, TCI, 98%) in anhydrous propylene carbonate (PC, Sigma-Aldrich, anhydrous, 99.7%). The TMA^+ electrolyte concentration was 0.1 M (TMA·BF_4 in PC), the TPA^+ electrolyte was 0.1 M (TPA·BF_4 in PC), and the hybrid electrolyte consisted of the concentrations for each salt were 0.1 M (TPA·BF_4) and 0.01 M (TMA·BF_4), respectively. An external electrochemical potential (-2 V vs. Ag) was applied for 30 min to facilitate the intercalation. To this end, the entire setup was kept in an inert atmosphere to prevent the oxidation of WTe_2. After the completion of intercalation, WTe_2 was exfoliated into nanoflakes, transferred into a centrifuge tube, and sealed with parafilm to prevent oxidation. Exfoliated WTe_2 flakes were washed thoroughly with anhydrous propylene carbonate and N,N-Dimethylformamide (DMF, Sigma-Aldrich, anhydrous, 99.8%), 3 times respectively, using centrifugation (5100 rpm, 5 min). Sedimented WTe_2 flakes were ultimately dispersed in 20 ml of DMF, and bath sonication was conducted for 15 min under an ice bath. The as-prepared WTe_2 dispersion was centrifuged at 3000 rpm for 5 min to separate unexfoliated and thick flakes from exfoliated material. To this end, the supernatant was collected for subsequent characterization and device fabrication. The exfoliation yield was calculated using the following equation: Exfoliation Yield (%) = (Mass of exfoliated WTe_2/Initial mass of bulk WTe_2) × 100.

was

Single-flake devices based on WTe_2 flakes and measurements

WTe_2 single-flake devices (10 nm thick) were fabricated in an argon atmosphere using p-doped silicon substrates covered with a 285 nm-thick thermally grown SiO_2. For this purpose, 150 μL of WTe_2 dispersion was spin-coated at 2000 rpm for 60 s at a 0.10 g/L concentration in DMF. Fabricated samples

were annealed at 200°C for 10 hours under argon atmosphere to remove residual solvent. The electrical and magnetic transport properties of the layered WTe_2 flakes were measured without and with applied magnetic field at various temperatures in a PPMS DynaCool cryostat (Quantum Design) with a base temperature of 1.8 K and a magnetic field up to 9 T. The magnetoresistance, expressed as MR (%), is calculated using the formula: MR (%) = (R_{xx} (B) - R_{xx} (0)) / R_{xx} (0)) ×100, where R_{xx} (B) represents the longitudinal resistance with an applied magnetic field in the out-of-plane direction, while the current is applied along the a-axis. R_{xx} (0) is the resistance measured in a zero field.

Using a two-band model ($MR\,(B) = \frac{\rho_{xx}(B) - \rho_{xx}(0)}{\rho_{xx}(0)} \approx {\mu_{avg}}^2 B^2$) and electron-hole compensation assumption (*e.g.*, $n_e \approx n_h = n$), the carrier mobility and concentration were extracted from measured data.[1–4] μ_{avg} is the average mobility under the condition of electron-hole compensation. At zero magnetic field, under the assumption of electron-hole compensation, the zero-field conductivity is the sum of electron and hole contributions: $\sigma_{xx}(0) \approx ne\mu_e + ne\mu_h \approx ne(\mu_e + \mu_h) \approx 2ne\mu_{avg}$. Assuming that μ_e and μ_h are of similar magnitude, average mobility becomes $\mu_{avg} = \frac{\mu_e + \mu_h}{2}$. Then, resistivity $\rho_{xx}(0)$ is expressed as: $\rho_{xx}(0) = \frac{1}{\sigma_{xx}(0)} = \frac{1}{2ne\mu_{avg}}$. Resistivity at 2 K, 0T has been calculated as $\rho_{xx}(0) \approx 2.306 \times 10^{-6}\ \Omega \cdot \mathrm{m}$.

From MR measurement data (*i.e.*, MR (9 T) ≈ 0.50), μ_{avg} was obtained using ${\mu_{avg}}^2 = \frac{MR(B)}{B^2}$.

The average carrier mobility (μ_{avg}) was approximately 785 cm^2/V·s.

The carrier concentration (n) was extracted by using $n = \frac{1}{2\rho_{xx}(0)e\mu_{avg}}$ and substituting each value. The carrier concentration (n) was approximately $1.72 \times 10^{19}\ cm^{-3}$.

WTe_2 large-area thin film deposition

For larger area homogeneous thin film deposition, a larger mass of nanosheets at high concentration was required, which causes problems with colloidal stability. To account for this, a large crystal of WTe_2 (~2 mg) was used for the initial exfoliation. After electrochemical exfoliation, 0.2 g/L poly(vinylpyrrolidone) (PVP) (molecular weight ~40000, Sigma-Aldrich) in DMF was added to the intercalated crystal to facilitate the exfoliation at high concentration while maintaining the colloidal

stability. In further detail, the intercalated crystal was immersed in PVP/DMF (0.2 g/L) solution and was bath-sonicated for 5 minutes. Thicker nanomaterials were removed by centrifugation at 991 rpm for 5 minutes. The resulting dispersion was washed in 4 steps to remove excess polymer and other impurities, including defective materials. For this purpose, we precipitate nanomaterials by centrifugation at 5100 rpm for 2 hours, remove the supernatant, and redisperse nanomaterials in the fresh solvent. This step was repeated 2x using DMF and 2x using IPA.
The resulting ink (c = 0.6 g/L) was used to study their thin film characteristics after a Langmuir-type deposition approach. The Langmuir-type approach was used to prepare tiled thin films with conformal inter-nanosheet junctions. The deposition process and custom-made device used for thin film fabrication are described in detail elsewhere.[5] However, due to the sensitivity of WTe_2 nanosheets to the ambient environment, the deposition was performed in an Argon atmosphere, using degassed water and hexane to form a liquid/liquid interface for the material injection. The Argon atmosphere was maintained in a tabletop glovebox (MB-Acryl GB 2202-P-VAC), and the oxygen content was kept below 0.1% (Greisinger O_2 analyzer, Accuracy:< 2 vol.% O_2: +/-0.2%).

Fabricated films were pre-dried at 50°C in an argon atmosphere for 15 minutes, and residual solvent was removed by increasing the temperature to 130°C for an additional 15 minutes. This enables the repetition of the deposition process on the same substrate as it improves the adhesion of the deposited nanosheet layer on the substrate. For electrical characterization, 3 depositions were performed, yielding a homogenous nanosheet network over the entire substrate area (225 mm^2) with an average thickness of 8.6 nm ± 0.8 nm. To study the ferroelectric properties of the exfoliated materials, PFM was used with dual frequency resonance tracking mode. To this end, separated flakes are required, which can easily be deposited by spin-coating. To this end, samples were prepared by spin-coating a thin layer of polystyrene on a gold-coated Si/SiO_2 wafer (Evaporated Cr/Au thickness: 10 nm/60 nm), using the following parameters (4000 rpm, 2 min, 2 wt% of polystyrene in Toluene, annealing at 150°C and then cooling at room temperature). The polymer solution was filtered using a 0.2 µm filter (PFTE-Membrane filter, fisher brand) to account for polymer bundles present in the suspension. Following this protocol, polystyrene film had the average thickness of 77.8 nm ± 2.7 nm (Figure S11). Diluted WTe_2 dispersion (c = 0.18 g/L) was spin-coated at 1800 rpm for 30 seconds, then annealed at 150°C overnight in an argon

atmosphere to prevent the degradation of WTe_2. Following this, a homogenous coverage of individual nanosheets was achieved over the entire substrate surface, suitable for the intended measurements. In addition to PFM switching spectroscopy on WTe_2 flakes, similar measurements were performed on bare polystyrene/gold substrates, which served as reference measurements.

Material Characterization

The WTe_2 flakes were characterized by a ZEISS Sigma field-emission scanning electron microscopy (FESEM), a Carl Zeiss Libra 200 high-resolution transmission electron microscopy (TEM), and Parksystems NX10 atomic force microscopy (AFM). For AFM statistical analysis, a total of 104 flakes were analyzed when using the TPA^+ electrolyte, and 193 flakes were analyzed for the hybrid electrolyte (TPA^+-TMA^+). Raman spectra were recorded using a WITEC alpha 300R Confocal Raman system fitted with a 532 nm excitation laser. Powder X-ray diffraction (XRD) data was collected via a Malvern PANalytical Aeris research edition powder diffractometer with Cu Kα radiation. X-ray photoelectron spectroscopy (XPS) measurements were performed using a K-Alpha X-ray Photoelectron Spectrometer System (Thermo Fisher Scientific) with a monochromatic X-ray source (Al K_α) with a spot diameter of 400 μm and an electron detector with an energy resolution of 0.5 eV. Charge compensation during data acquisition was performed by the internal electron flood gun of the system. The spectra were calibrated using the C 1s peak (284.6 eV) and fitted using Voigt functions after background subtraction. Piezoresponse force microscopy (PFM) was measured with Asylum Research (Oxford instruments) MFP3D AFM in dual amplitude resonance tracking (DART) PFM with an AC oscillation amplitude of 3 V at 330 kHz initial resonance frequency (local contact resonance frequency) with the sidebands placed 3 kHz above and below the resonance (327 and 333 kHz). An additional frequency feedback compares the amplitudes of the PFM response at these two frequencies and adjusts the excitation frequencies in order to keep the amplitude ratio constant in real time, thereby adjusting to the local contact stiffness and surface topography. By monitoring the amplitude and phase responses at these two frequencies, DART ensures that the measurements remain near the resonance condition,[6,7] resulting in: enhanced detection sensitivity to weak piezoresponse signals via resonance enhancement, reduced crosstalk from surface topography and mechanical artifacts, and improved stability in measurements on

soft or structurally inhomogeneous materials. The tracked contact resonance featured a shift from the substrate to the flake, corresponding to a successfully compensated change in contact mechanics (Figure S14). The used cantilever was Budget Sensors ElectriMulti75-G with PtIr coating. Switching spectroscopy was measured at a sweep rate of 0.1 Hz over 4 cycles.

Computational Details

All density functional theory (DFT) based calculations were carried out using projected augmented wave (PAW)[8] pseudopotentials as implemented in the Vienna Ab-initio Simulation Package (VASP).[9,10] The Kohn-Sham orbitals were expanded in the plane-wave basis with a cutoff of 550 eV. optB86b exchange[11] plus PBE correlation[12] together with optB86b-vdW were employed to describe the exchange-correlation and van der Waals interactions. The structures were optimized until the force components were less than 0.1 eV/ Å. Calculations were performed for a 6×3 cell with various layers of WTe_2 to simulate different stages. Brillouin zone integration was performed with a Γ-centered k-point mesh. The powder diffraction pattern is calculated and visualized by VESTA.[13]

Supporting information

Table S1. A summary of theoretical diffraction patterns for different cations and intercalation stages.

Cations	Stage	d_{PDP} (Å)
TMA	8	7.2
TMA	6	7.2
TMA	4	7.4
TMA	3	7.5
TMA	2	8.02
TMA	1	11.15

Cations	Stage	d_{PDP} (Å)
TPA	8	12.6, 9.0, 7.0
TPA	6	12.2, 9.8, 7.0
TPA	4	11.3, 8.5, 6.8
TPA	3	13.5, 9.0, 6.7
TPA	2	9.9, 6.6
TPA	1	14.2

Cations	Stage	d_{PDP} (Å)
TMA	6	7.2
TPA	8	12.6, 9.0, 7.0
TPA+TMA	3	12.4, 9.9, 8.3, 7.1

Table S2. A summary of electrochemically exfoliated MoS_2 and $MoSe_2$.

Material	Intercalant	Lateral size	Thickness	Reference
MoS_2	Tetraheptylammonium	0.5–2 µm	3.8 nm	[14]
MoS_2	Tetraheptylammonium	0.58 µm	1.8 nm	[15]
MoS_2	Tetraheptylammonium	1.5 µm	1.6 nm	[16]
MoS_2	Tetrapropylammonium + Tetramethylammonium	3.78 µm	2.16 nm	This work
$MoSe_2$	Tetraheptylammonium	-	-	[17]
$MoSe_2$	Tetrahexylammonium	-	-	[18]
$MoSe_2$	Tetrapropylammonium + Tetramethylammonium	3.63 µm	1.95 nm	This work

Table S3. A summary of different methods to achieve 2D WTe_2.

Methods	Size (µm)	Thickness	Time	Solution-processable (Yes/ No)	Reference
Electrochemical exfoliation	~ 2	1 – 3 layers	30 min	Y	This work
Chemical vapor deposition	~ 350	Monolayer	> 1 h	N	[19]
Liquid phase exfoliation	< 1	2 – 7 layers	8 h	Y	[20]
Mechanical exfoliation	~ 30	Few layers	> 1 h	N	[21]
Molecular beam epitaxy	0.02 – 0.03	Monolayer	8.5 h	N	[22]

Table S4. Reported magnetoresistance of WTe_2 via different preparation methods.

Methods	Thickness (nm)	Temperature (K)	Magnetic field (T)	Magnetoresistance (%)	Reference
Electrochemical exfoliation	10	2	9	~ 50	This work
Mechanical exfoliation	10	0.03	10	~ 80	[23]
Mechanical exfoliation	13	1.8	9	~ 160	[2]
Chemical vapor deposition	9.4	2	9	~ 3	[24]

Table S5. The conductivity of solution-processed 2D materials thin films.

Methods	Material	Deposition process	Thickness of thin film	Conductivity (S/m)	Reference
Electrochemical exfoliation	WTe_2	Langmuir-Schaefer method	8.6 nm	2.9×10^4	This work
Liquid phase exfoliation	$PtSe_2$	Langmuir-Blodgett method	241 nm	7.0×10^2	[25]
Liquid phase exfoliation	Graphene	Spray coating	500 nm	1.2×10^5	[26]
Liquid phase exfoliation	Graphene	Screen printing	7800 nm	7.1×10^4	[27]
Electrochemical exfoliation	Graphene	Vacuum filtration	50 nm	5.9×10^4	[28]
Electrochemical exfoliation	Graphene	Langmuir-Schaefer method	11 nm	1.3×10^5	[29]
Liquid phase exfoliation	MgB_2	Langmuir-Schaefer method	570 nm	9.5×10^4	[30]

Figure S1. a) SEM image, b) AFM image of exfoliated WTe_2 flakes using TPA^+ electrolyte.

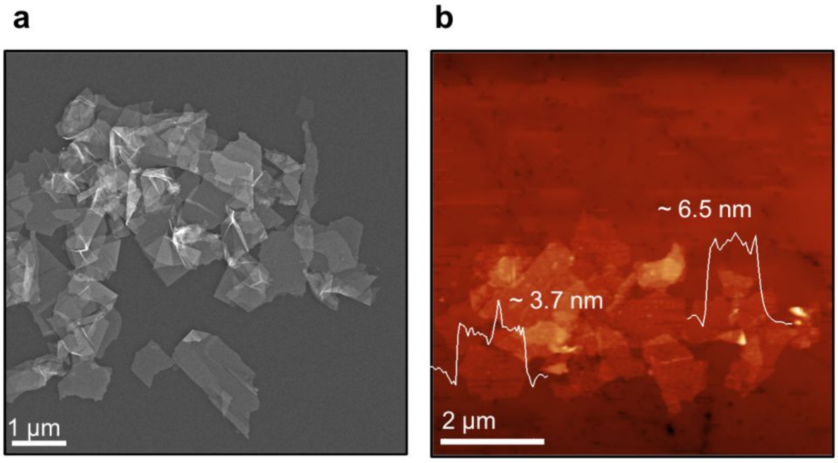

Figure S2. Simulated intercalation stages for TPA^+ intercalation. a) stage 1, b) stage 2, c) stage 3, d) stage 4, e) stage 6, and f) stage 8.

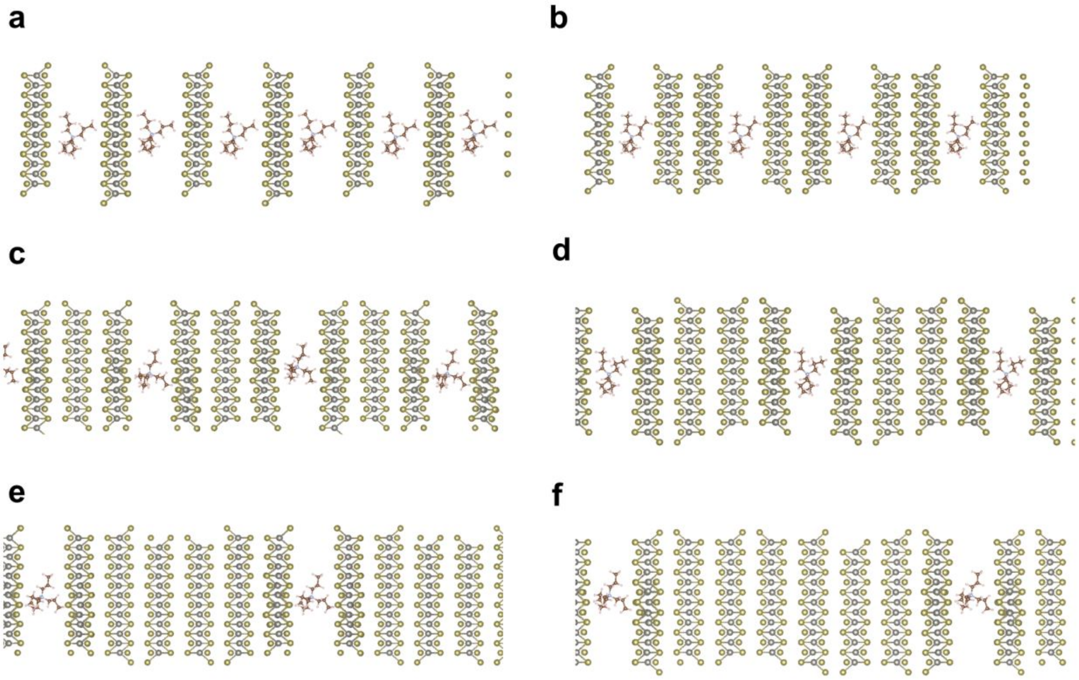

Figure S3. Simulated intercalation stages for TMA^+ intercalation. a) stage 1, b) stage 2, c) stage 3, d) stage 4, e) stage 5, and f) stage 6.

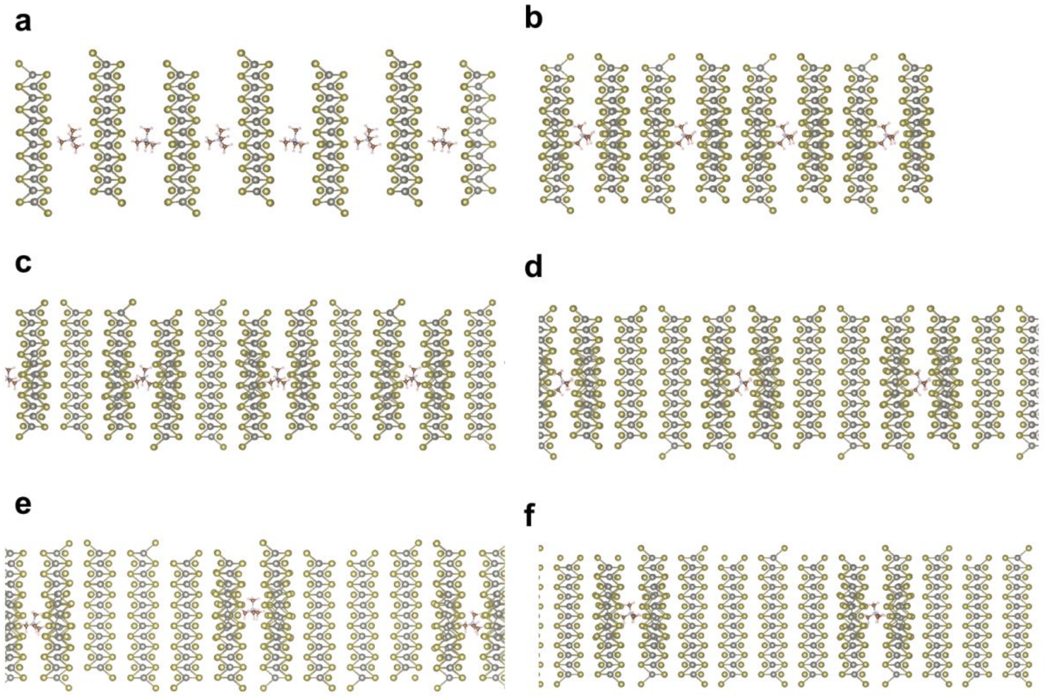

Figure S4. Cyclic voltammetry analysis for different electrolytes over the cycles.

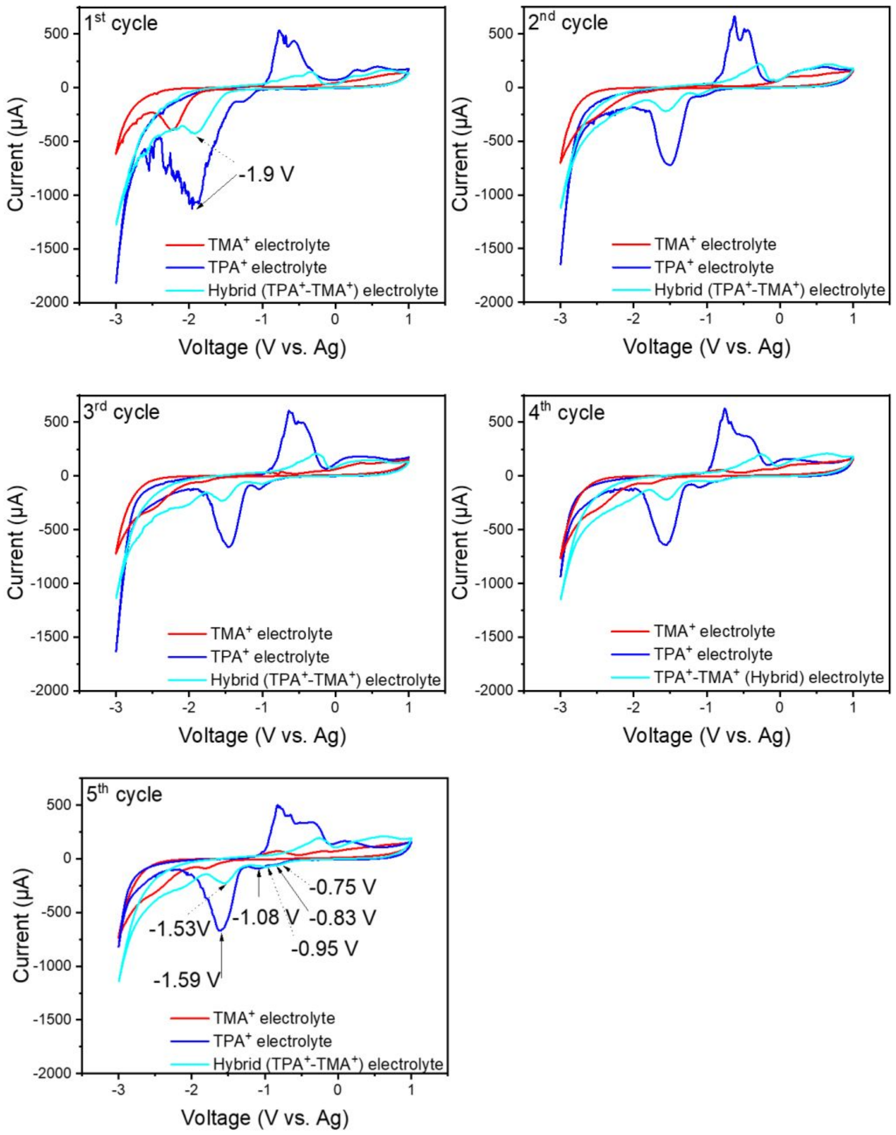

Figure S5. Cyclic voltammetry measurements for different electrolytes in the range from -3 V to 1 V with a scan rate of 10 mV/s at 5th cycle, a) 0.1 M TMA·BF_4, b) 0.1 M TEA·BF_4, c) 0.1 M TPA·BF_4, d) 0.1 M TPA·BF_4 + 0.1 M TMA·BF_4, e) 0.1 M TPA·BF_4 + 0.05 M TMA·BF_4, f) 0.1 M TPA·BF_4 + 0.02 M TMA·BF_4, g) 0.1 M TPA·BF_4 + 0.01 M TMA·BF_4.

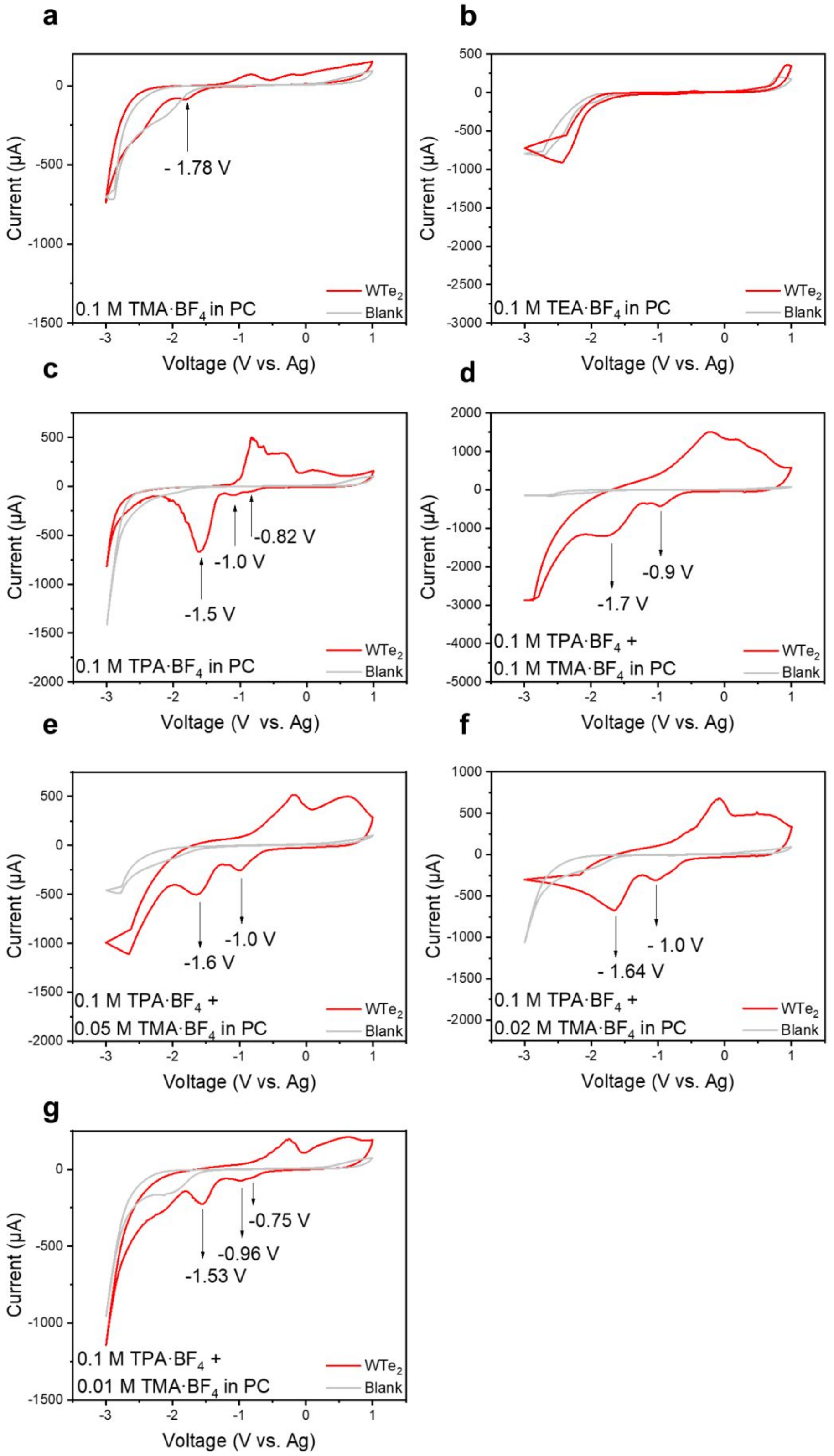

Figure S6. Distribution of relaxation time and Nyquist plot for different electrolytes (TMA^+, TPA^+, hybrid (TPA^+-TMA^+)) before and after the exfoliation process.

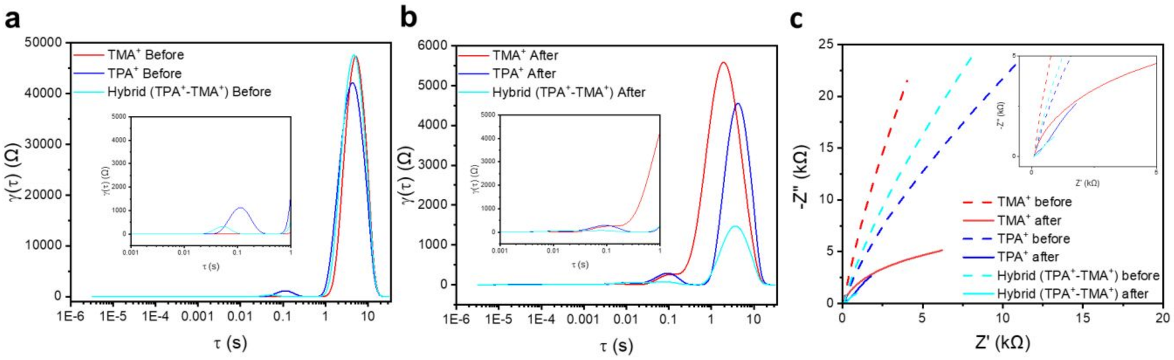

Figure S7. SEM image of a large WTe_2 flake from electrochemical exfoliation.

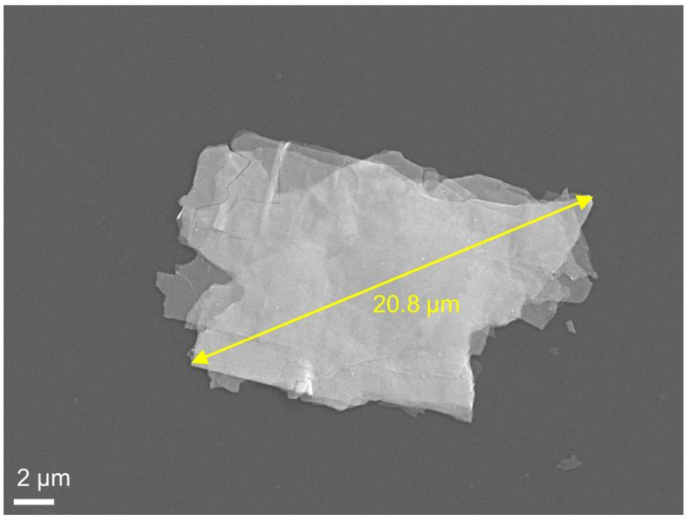

Figure S8. The delamination yields for different concentrations of electrolytes and different working biases.

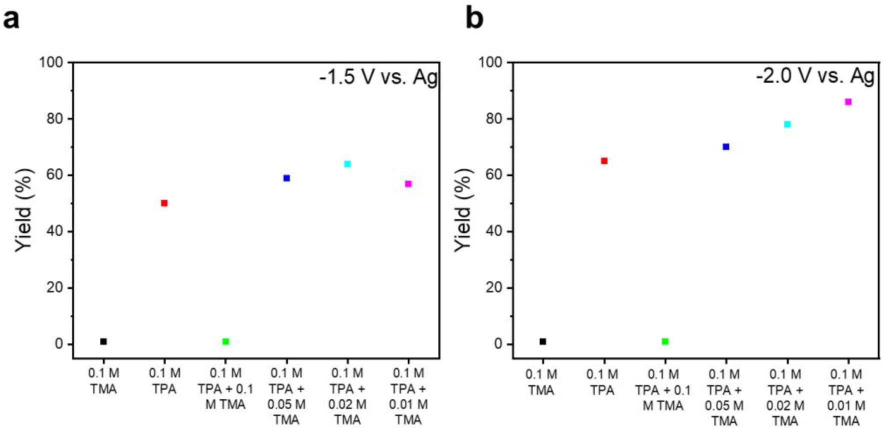

Figure S9. a), b) Lateral size distribution, c), d) Thickness distribution of exfoliated MoS_2 and $MoSe_2$ flakes (normalized) obtained from TPA^+ electrolyte and hybrid electrolyte.

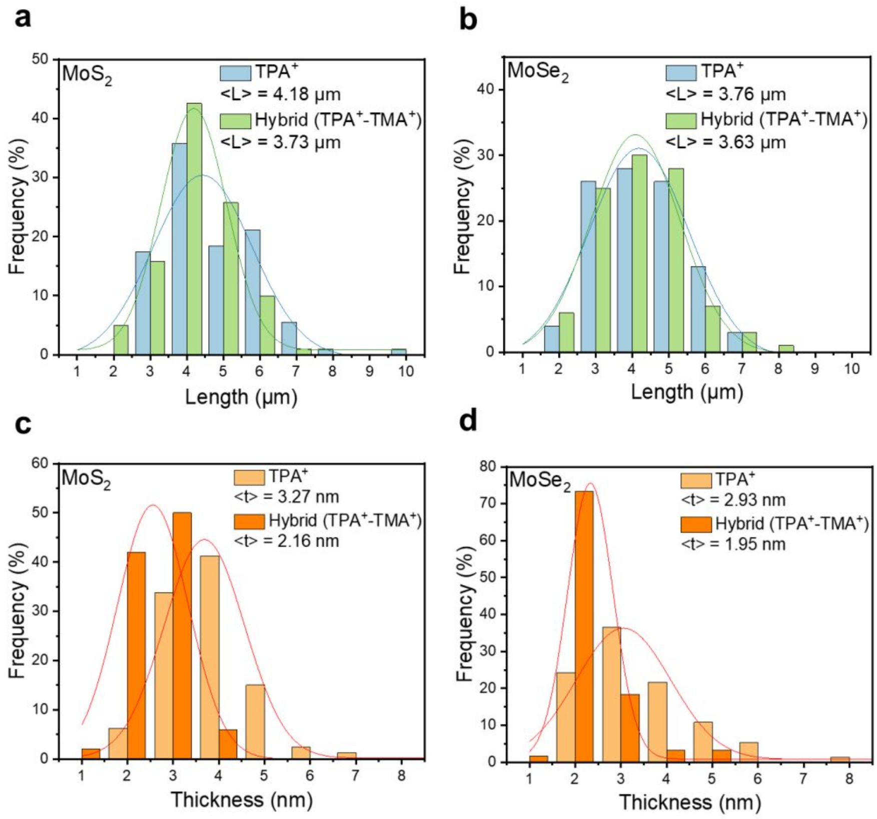

Figure S10. HR-TEM of WTe_2 flakes.

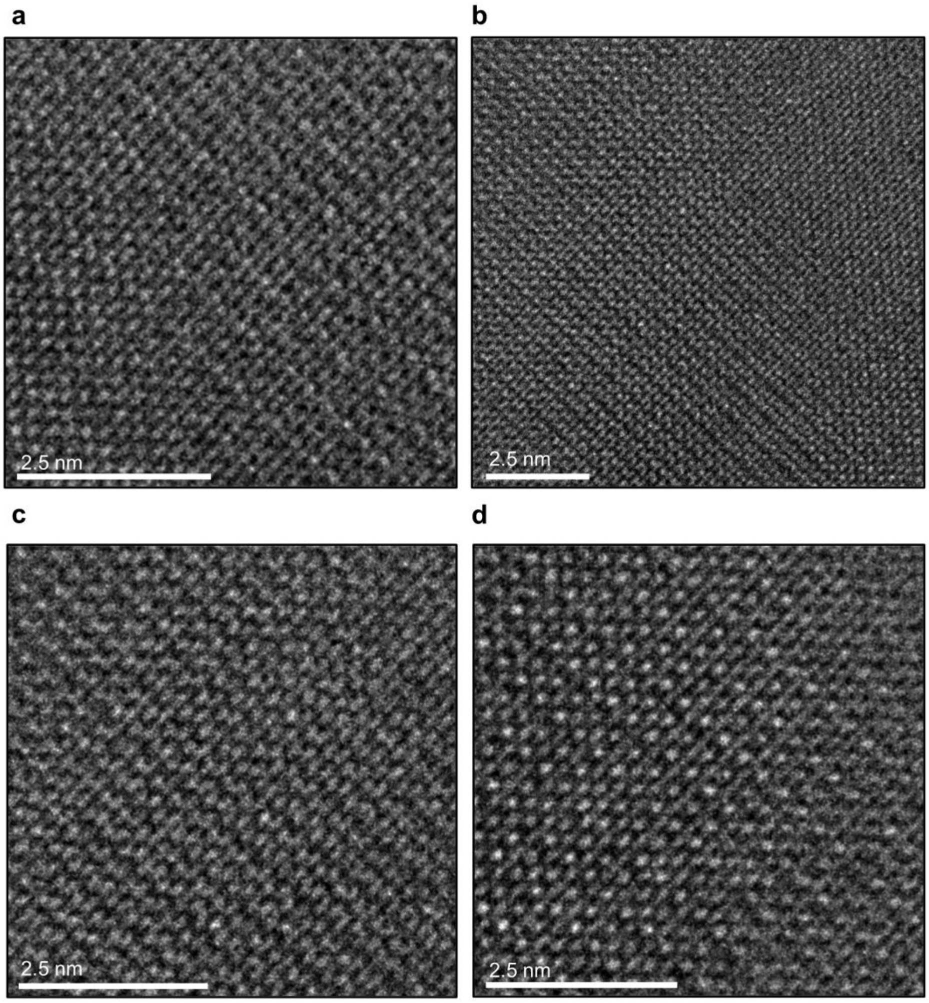

Figure S11. XPS of WTe_2 crystals and WTe_2 flakes.

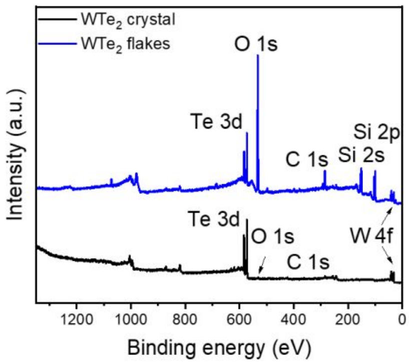

Figure S12. AFM images of WTe_2 flake exposed in the air for 14 days. The scale bar is 400 nm.

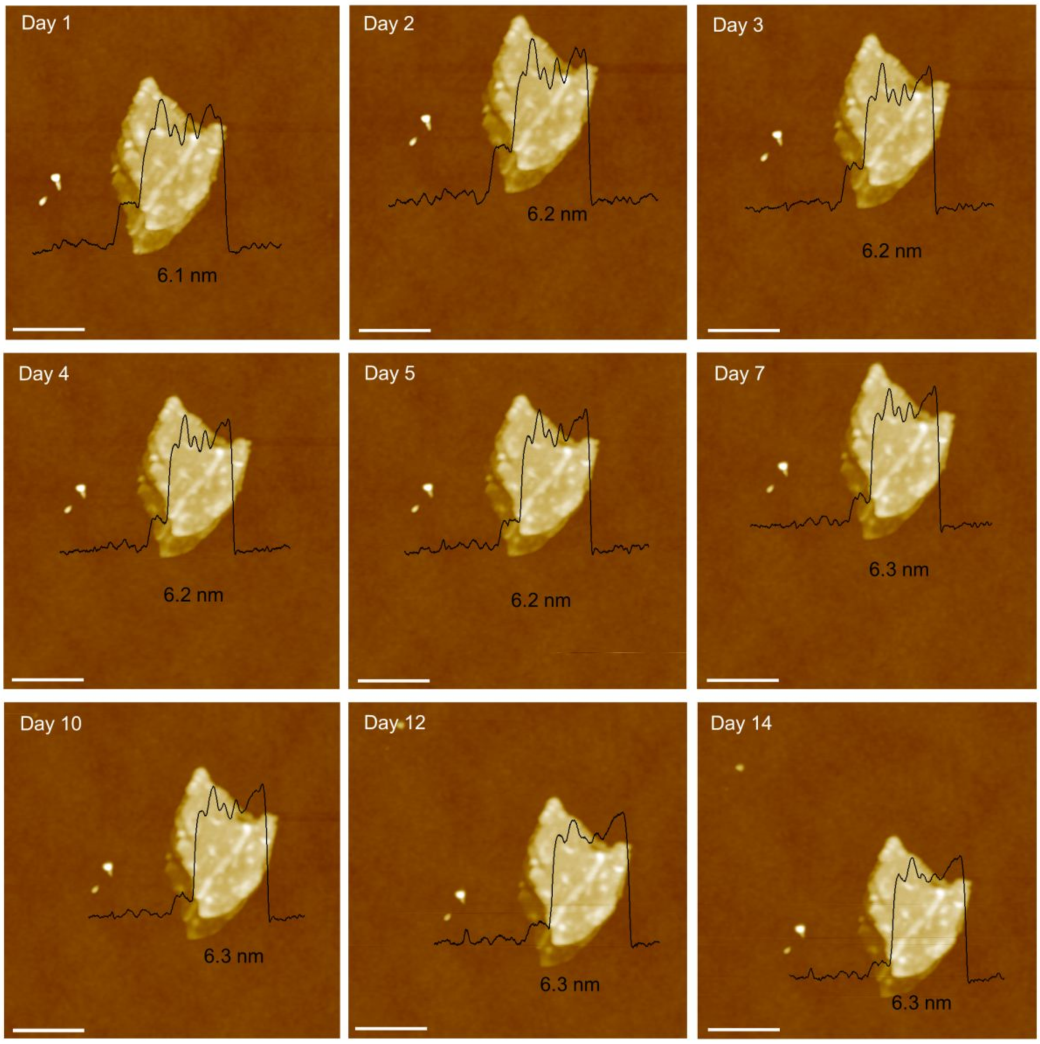

Figure S13. a) AFM image of polystyrene/gold substrate. b) thickness of polystyrene film on top of gold substrate.

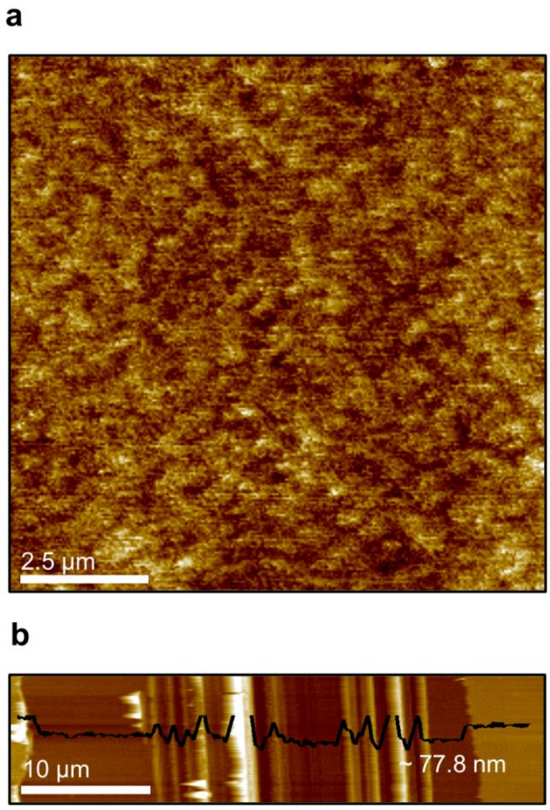

Figure S14. Dual amplitude resonance tracking (DART) PFM image of WTe_2 flake on a gold substrate. a) topography, b) phase, c) amplitude, d) contact resonance frequency.

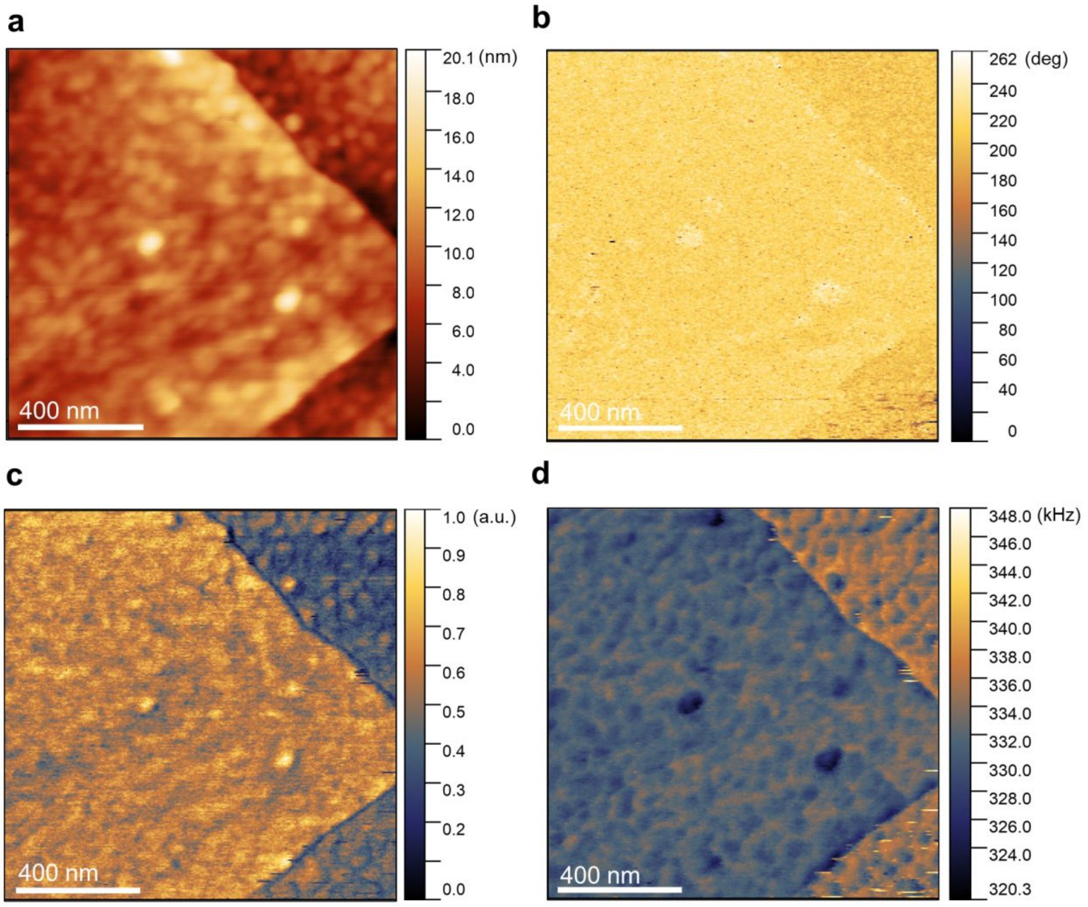

Figure S15. a) AFM topography of WTe_2 flakes on the polystyrene/gold substrate. b) PFM amplitude butterfly loops, c) PFM phase hysteresis loops on different positions on WTe_2 flakes.

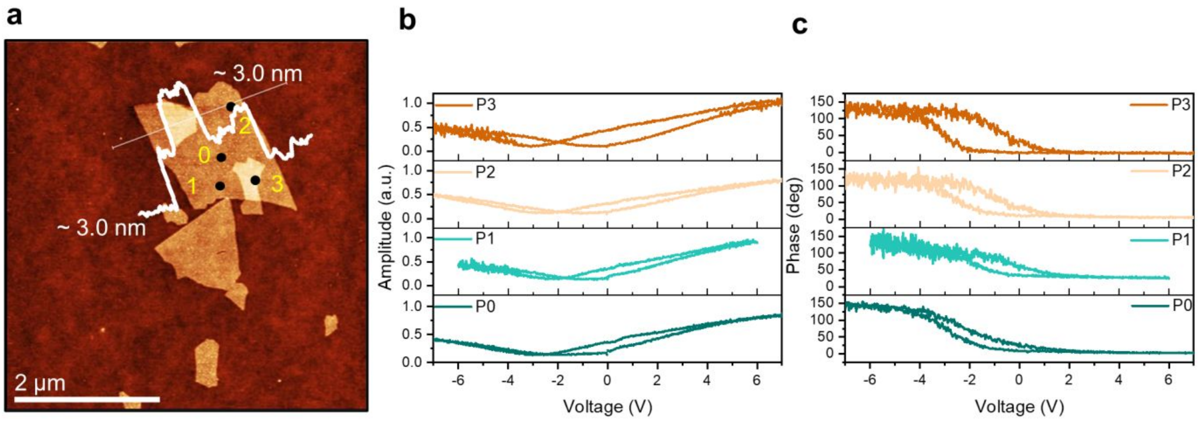

References

(1) Wang, L.; Gutiérrez-Lezama, I.; Barreteau, C.; Ubrig, N.; Giannini, E.; Morpurgo, A. F. Tuning Magnetotransport in a Compensated Semimetal at the Atomic Scale. *Nat. Commun.* **2015**, *6*, 1–7. https://doi.org/10.1038/ncomms9892.

(2) Woods, J. M.; Shen, J.; Kumaravadivel, P.; Pang, Y.; Xie, Y.; Pan, G. A.; Li, M.; Altman, E. I.; Lu, L.; Cha, J. J. Suppression of Magnetoresistance in Thin WTe 2 Flakes by Surface Oxidation. *ACS Appl. Mater. Interfaces* **2017**, *9* (27), 23175–23180. https://doi.org/10.1021/acsami.7b04934.

(3) Ali, M. N.; Xiong, J.; Flynn, S.; Tao, J.; Gibson, Q. D.; Schoop, L. M.; Liang, T.; Haldolaarachchige, N.; Hirschberger, M.; Ong, N. P.; Cava, R. J. Large, Non-Saturating Magnetoresistance in WTe 2. *Nature* **2014**, *514* (7521), 205–208. https://doi.org/10.1038/nature13763.

(4) Das, P. K.; Di Sante, D.; Cilento, F.; Bigi, C.; Kopic, D.; Soranzio, D.; Sterzi, A.; Krieger, J. A.; Vobornik, I.; Fujii, J.; Okuda, T.; Strocov, V. N.; Breese, M. B. H.; Parmigiani, F.; Rossi, G.; Picozzi, S.; Thomale, R.; Sangiovanni, G.; Cava, R. J.; Panaccione, G. Electronic Properties of Candidate Type-II Weyl Semimetal WTe2. A Review Perspective. *Electronic Structure* **2019**, *1* (1). https://doi.org/10.1088/2516-1075/ab0835.

(5) Carey, T.; Cassidy, O.; Synnatschke, K.; Caffrey, E.; Garcia, J.; Liu, S.; Kaur, H.; Kelly, A. G.; Munuera, J.; Gabbett, C.; O'Suilleabhain, D.; Coleman, J. N. High-Mobility Flexible Transistors with Low-Temperature Solution-Processed Tungsten Dichalcogenides. *ACS Nano* **2023**, *17* (3), 2912–2922. https://doi.org/10.1021/acsnano.2c11319.

(6) Gannepalli, A.; Yablon, D. G.; Tsou, A. H.; Proksch, R. Mapping Nanoscale Elasticity and Dissipation Using Dual Frequency Contact Resonance AFM. *Nanotechnology* **2013**, *24* (15), 159501. https://doi.org/10.1088/0957-4484/24/15/159501.

(7) Rodriguez, B. J.; Callahan, C.; Kalinin, S. V.; Proksch, R. Dual-Frequency Resonance-Tracking Atomic Force Microscopy. *Nanotechnology* **2007**, *18* (47), 475504. https://doi.org/10.1088/0957-4484/18/47/475504.

(8) Blöchl, P. E. Projector Augmented-Wave Method. *Phys. Rev. B* **1994**, *50* (24), 17953–17979. https://doi.org/10.1103/PhysRevB.50.17953.

(9) Furthmüller, G. K. and J. Efficient Iterative Schemes for Ab Initio Total-Energy Calculations Using a Plane-Wave Basis Set. *Phys. Rev. B* **1996**, *54* (16), 11169–11186. https://doi.org/10.1103/PhysRevB.54.11169.

(10) Kresse, G.; Hafner, J. Ab Initio Molecular Dynamics for Liquid Metals. *Phys. Rev. B* **1993**, *47* (1), 558–561. https://doi.org/10.1103/PhysRevB.47.558.

(11) Klime, J.; Bowler, D. R.; Michaelides, A. Van Der Waals Density Functionals Applied to Solids. *Phys. Rev. B* **2011**, *83* (19), 195131. https://doi.org/10.1103/PhysRevB.83.195131.

(12) Perdew, J. P.; Burke, K.; Ernzerhof, M. Generalized Gradient Approximation Made Simple. *Phys. Rev. Lett.* **1997**, *77* (18), 3865–3868. https://doi.org/10.1103/PhysRevLett.77.3865.

(13) Momma, K.; Izumi, F. VESTA 3 for Three-Dimensional Visualization of Crystal, Volumetric and Morphology Data. *J Appl. Crystallogr.* **2011**, *44* (6), 1272–1276. https://doi.org/10.1107/S0021889811038970.

(14) Lin, Z.; Liu, Y.; Halim, U.; Ding, M.; Liu, Y.; Wang, Y.; Jia, C.; Chen, P.; Duan, X.; Wang, C.; Song, F.; Li, M.; Wan, C.; Huang, Y.; Duan, X. Solution-Processable 2D Semiconductors for

High-Performance Large-Area Electronics. *Nature* **2018**, *562* (7726), 254–258. https://doi.org/10.1038/s41586-018-0574-4.

(15) Carey, T.; Arbab, A.; Anzi, L.; Bristow, H.; Hui, F.; Bohm, S.; Wyatt-Moon, G.; Flewitt, A.; Wadsworth, A.; Gasparini, N.; Kim, J. M.; Lanza, M.; McCulloch, I.; Sordan, R.; Torrisi, F. Inkjet Printed Circuits with 2D Semiconductor Inks for High-Performance Electronics. *Adv. Electron. Mater* **2021**, *7*, 2100112. https://doi.org/10.1002/aelm.202100112.

(16) Kong, L.; Li, G.; Su, Q.; Zhang, X.; Liu, Z.; Liao, G.; Sun, B.; Shi, T. Inkjet-Printed, Large-Area, Flexible Photodetector Array Based on Electrochemical Exfoliated MoS2 Film for Photoimaging. *Adv. Eng. Mater.* **2023**, *25* (2), 2200946. https://doi.org/10.1002/adem.202200946.

(17) Zou, T.; Kim, H. J.; Kim, S.; Liu, A.; Choi, M. Y.; Jung, H.; Zhu, H.; You, I.; Reo, Y.; Lee, W. J.; Kim, Y. S.; Kim, C. J.; Noh, Y. Y. High-Performance Solution-Processed 2D P-Type WSe2 Transistors and Circuits through Molecular Doping. *Adv. Mater.* **2023**, *35* (7), 2208934. https://doi.org/10.1002/adma.202208934.

(18) Yu, W.; Dong, Z.; Abdelwahab, I.; Zhao, X.; Shi, J.; Shao, Y.; Li, J.; Hu, X.; Li, R.; Ma, T.; Wang, Z.; Xu, Q. H.; Tang, D. Y.; Song, Y.; Loh, K. P. High-Yield Exfoliation of Monolayer 1T'-MoTe2as Saturable Absorber for Ultrafast Photonics. *ACS Nano* **2021**, *15* (11), 18448–18457. https://doi.org/10.1021/acsnano.1c08093.

(19) Chen, K.; Chen, Z.; Wan, X.; Zheng, Z.; Xie, F.; Chen, W.; Gui, X.; Chen, H.; Xie, W.; Xu, J. A Simple Method for Synthesis of High-Quality Millimeter-Scale 1T′ Transition-Metal Telluride and Near-Field Nanooptical Properties. *Adv. Mater.* **2017**, *29* (38), 1700704. https://doi.org/10.1002/adma.201700704.

(20) Yu, P.; Fu, W.; Zeng, Q.; Lin, J.; Yan, C.; Lai, Z.; Tang, B.; Suenaga, K.; Zhang, H.; Liu, Z. Controllable Synthesis of Atomically Thin Type-II Weyl Semimetal WTe2 Nanosheets: An Advanced Electrode Material for All-Solid-State Flexible Supercapacitors. *Adv. Mater.* **2017**, *29* (34), 1701909. https://doi.org/10.1002/adma.201701909.

(21) Wang, Y.; Liu, E.; Liu, H.; Pan, Y.; Zhang, L.; Zeng, J.; Fu, Y.; Wang, M.; Xu, K.; Huang, Z.; Wang, Z.; Lu, H. Z.; Xing, D.; Wang, B.; Wan, X.; Miao, F. Gate-Tunable Negative Longitudinal Magnetoresistance in the Predicted Type-II Weyl Semimetal WTe 2. *Nat. Commun.* **2016**, *7*, 13142. https://doi.org/10.1038/ncomms13142.

(22) Maximenko, Y.; Chang, Y.; Chen, G.; Hirsbrunner, M. R.; Swiech, W.; Hughes, T. L.; Wagner, L. K.; Madhavan, V. Nanoscale Studies of Electric Field Effects on Monolayer 1T′-WTe2. *npj Quantum Mater.* **2022**, *7* (1), 29. https://doi.org/10.1038/s41535-022-00433-x.

(23) Xiang, F.-X.; Srinivasan, A.; Du, Z. Z.; Klochan, O.; Dou, S.-X.; Hamilton, A. R.; Wang, X.-L. Thickness-Dependent Electronic Structure in WTe2 Thin Films. *Phys. Rev. B* **2018**, *98* (3), 035115. https://doi.org/10.1103/PhysRevB.98.035115.

(24) Lin, E.-C.; Lin, Y.-T.; Chou, C.-T.; Chen, C.-A.; Wu, Y.-J.; Chen, P.-H.; Lee, S.-F.; Chang, C.-S.; Chen, Y.-F.; Lee, Y.-H. Enhanced Magnetoresistance of Doped WTe 2 Single Crystals. *ACS Appl. Electron. Mater.* **2022**, *4* (9), 4540–4546. https://doi.org/10.1021/acsaelm.2c00763.

(25) Lee, K.; Szydłowska, B. M.; Hartwig, O.; Synnatschke, K.; Tywoniuk, B.; Hartman, T.; Tomašević-Ilić, T.; Gabbett, C. P.; Coleman, J. N.; Sofer, Z.; Spasenović, M.; Backes, C.; Duesberg, G. S. Highly Conductive and Long-Term Stable Films from Liquid-Phase Exfoliated Platinum Diselenide. *J Mater. Chem. C.* **2023**, *11* (2), 593–599. https://doi.org/10.1039/d2tc03889g.

(26) Clifford, K.; Ogilvie, S. P.; Amorim Graf, A.; Wood, H. J.; Sehnal, A. C.; Salvage, J. P.; Lynch, P. J.; Large, M. J.; Dalton, A. B. Emergent High Conductivity in Size-Selected Graphene Networks. *Carbon* **2024**, *218*, 118642. https://doi.org/10.1016/j.carbon.2023.118642.

(27) Pan, K.; Fan, Y.; Leng, T.; Li, J.; Xin, Z.; Zhang, J.; Hao, L.; Gallop, J.; Novoselov, K. S.; Hu, Z. Sustainable Production of Highly Conductive Multilayer Graphene Ink for Wireless Connectivity and IoT Applications. *Nat. Commun.* **2018**, *9* (1), 5197. https://doi.org/10.1038/s41467-018-07632-w.

(28) Parvez, K.; Li, R.; Puniredd, S. R.; Hernandez, Y.; Hinkel, F.; Wang, S.; Feng, X.; Müllen, K. Electrochemically Exfoliated Graphene as Solution-Processable, Highly Conductive Electrodes for Organic Electronics. *ACS Nano* **2013**, *7* (4), 3598–3606. https://doi.org/10.1021/nn400576v.

(29) Cassidy, O.; Synnatschke, K.; Munuera, J. M.; Gabbett, C.; Carey, T.; Doolan, L.; Caffrey, E.; Coleman, J. N. Layer-by-Layer Assembly Yields Thin Graphene Films with near Theoretical Conductivity. *npj 2D Mater. Appl.* **2025**, *9* (2), 1–11. https://doi.org/10.1038/s41699-025-00525-9.

(30) Synnatschke, K.; Muller, A.; Gabbett, C.; Mohn, M. J.; Kelly, A. G.; Mosina, K.; Wu, B.; Caffrey, E.; Cassidy, O.; Backes, C.; Sofer, Z.; Kaiser, U.; Coleman, J. N. Inert Liquid Exfoliation and Langmuir-Type Thin Film Deposition of Semimetallic Metal Diborides. *ACS Nano* **2024**, *18* (42), 28596–28608. https://doi.org/10.1021/acsnano.4c04626.
